# Dual RNA Processing Roles of Pat1b via Cytoplasmic Lsm1-7 and Nuclear Lsm2-8 Complexes

**DOI:** 10.1016/j.celrep.2017.06.091

**Published:** 2017-08-01

**Authors:** Caroline Vindry, Aline Marnef, Helen Broomhead, Laure Twyffels, Sevim Ozgur, Georg Stoecklin, Miriam Llorian, Christopher W. Smith, Juan Mata, Dominique Weil, Nancy Standart

**Affiliations:** 1 Department of Biochemistry, University of Cambridge, Tennis Court Road, Cambridge CB2 1QW, UK; 2 LBCMCP, Centre de Biologie Intégrative (CBI), CNRS, Université de Toulouse UT3, 31062 Toulouse, France; 3 Center for Microscopy and Molecular Imaging (CMMI), Université libre de Bruxelles (ULB), 6041 Gosselies, Belgium; 4 Max Planck Institute of Biochemistry, Am Klopferspitz, 82152 Martinsried, Germany; 5 Division of Biochemistry, Center for Biomedicine and Medical Technology Mannheim, Medical Faculty Mannheim, Heidelberg University, 69047 Heidelberg, Germany; 6 Center for Molecular Biology of Heidelberg University (ZMBH), 69047 Heidelberg, Germany; 7 German Cancer Research Center (DKFZ), DKFZ-ZMBH Alliance, 68167 Mannheim, Germany; 8 Sorbonne Universités, UPMC Univ Paris 06, CNRS, Biologie du développement Paris Seine - Institut de Biologie Paris Seine (LBD - IBPS), 75005 Paris, France

**Keywords:** Pat1b, PATL1, Lsm, tri-snRNP, SART3, Prp31, P bodies, Cajal bodies, mRNA decay, alternative splicing

## Abstract

Pat1 RNA-binding proteins, enriched in processing bodies (P bodies), are key players in cytoplasmic 5′ to 3′ mRNA decay, activating decapping of mRNA in complex with the Lsm1-7 heptamer. Using co-immunoprecipitation and immunofluorescence approaches coupled with RNAi, we provide evidence for a nuclear complex of Pat1b with the Lsm2-8 heptamer, which binds to the spliceosomal U6 small nuclear RNA (snRNA). Furthermore, we establish the set of interactions connecting Pat1b/Lsm2-8/U6 snRNA/SART3 and additional U4/U6.U5 tri-small nuclear ribonucleoprotein particle (tri-snRNP) components in Cajal bodies, the site of snRNP biogenesis. RNA sequencing following Pat1b depletion revealed the preferential upregulation of mRNAs normally found in P bodies and enriched in 3′ UTR AU-rich elements. Changes in >180 alternative splicing events were also observed, characterized by skipping of regulated exons with weak donor sites. Our data demonstrate the dual role of a decapping enhancer in pre-mRNA processing as well as in mRNA decay via distinct nuclear and cytoplasmic Lsm complexes.

## Highlights

• Pat1b complexes with nuclear Lsm2-8 in addition to cytosolic Lsm1-7 heptamer
• Pat1b/Lsm2-8 binds U6 snRNA/SART3 and other tri-snRNP components in Cajal bodies
• Pat1b depletion stabilizes mRNA and promotes skipping of cassette exons in pre-mRNA
• Pat1b regulates distinct genes at the level of splicing and mRNA decay

## Graphical Abstract

**Figure:**
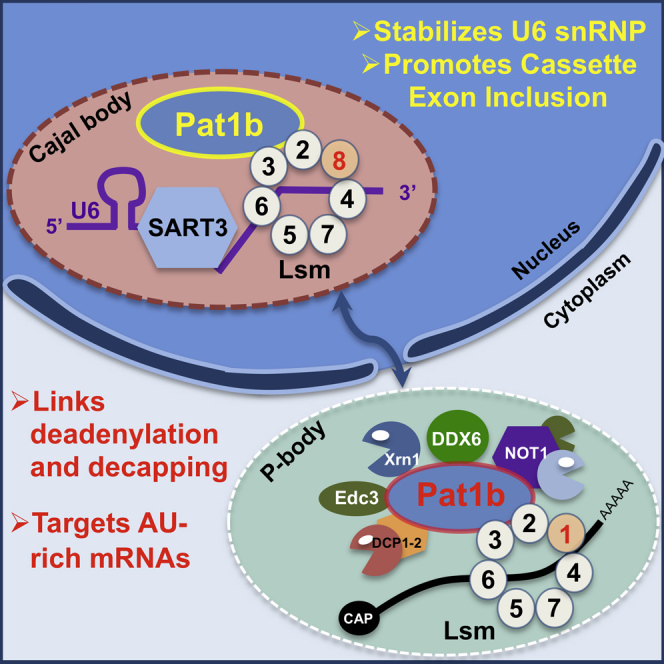

## Teaser

Pat1 RNA-binding proteins, enriched in P bodies, are key players in cytoplasmic 5′ to 3′ mRNA decay. Vindry et al. demonstrate an additional role of human Pat1b in alternative splicing via the nuclear Lsm complex and identify distinct mRNA targets of Pat1b-dependent splicing and decay regulation.

## Introduction

Pat1 RNA-binding proteins (DNA topoisomerase 2-associated protein; PATL1) are conserved from fungi to humans. Deletion of Pat1 results in a thermosensitive slow growth phenotype in *Saccharomyces cerevisiae* (Bonnerot et al., 2000, Wyers et al., 2000), dwarfism in *Arabidopsis thaliana* (Roux et al., 2015), and embryonic lethality in *Caenorhabditis elegans* and *Drosophila melanogaster* (Kamath et al., 2003, Pradhan et al., 2012). Moreover, homozygous PATL1 knockout mice are sub-viable (Dickinson et al., 2016). Such incomplete penetrance is often associated with paralog proteins. Indeed, while yeast and invertebrates possess one paralog, vertebrates have two: Pat1a (PATL2), which is expressed in oocytes, and Pat1b (PATL1), which is expressed in embryos and the soma (reviewed in Marnef and Standart, 2010).

Sequence conservation within and between Pat1 paralogs is largely confined to their C-terminal half portions, with the N-terminal half predicted to be largely disordered (Jonas and Izaurralde, 2013, Marnef and Standart, 2010). The C-terminal half region comprises the so-called Mid domain and the PatC domain, the only structurally solved domain that forms an α-α superhelix (Braun et al., 2010, Sharif and Conti, 2013, Wu et al., 2014). Pat1b and invertebrate orthologs function in cytoplasmic mRNA decay, as first shown in yeast, where its deletion stabilized reporter mRNA in a capped and deadenylated form (Bonnerot et al., 2000, Bouveret et al., 2000, Tharun et al., 2000). This led to its classification as an enhancer of decapping, as deadenylation precedes decapping in the 5′-to-3′ mRNA decay pathway. In *Drosophila* and mammalian tissue cells, its mRNA decay function has been largely evidenced by the tether function assay (Braun et al., 2010, Haas et al., 2010, Kamenska et al., 2014, Marnef and Standart, 2010, Ozgur et al., 2010, Totaro et al., 2011). Pat1 proteins also mediate translational repression in yeast and *Xenopus* oocytes (Coller and Parker, 2005, Marnef et al., 2010).

The multiple conserved roles of Pat1 proteins in mRNA silencing at the level of turnover and translation were affirmed in investigations of its binding partners. In yeast, the best-characterized interaction of Pat1p is with the Lsm1-7 complex. This cytoplasmic heptamer complexed to Pat1p tends to bind U-rich tracts at or near the 3′ end of oligoadenylated rather than polyadenylated mRNA (Chowdhury et al., 2007, Mitchell et al., 2013), protecting the last 20–30 nt of the transcript (He and Parker, 2001). These RNA-binding properties require both the Lsm1-7 ring and Pat1p (Chowdhury et al., 2014). Moreover, Lsm1-7 subunits and Pat1p are required for normal rates of decapping in vivo (Bouveret et al., 2000, Tharun et al., 2000). The Lsm1-7/Pat1 complex is thus considered as a conserved player in mRNA decay, linking deadenylation to decapping. Subsequent structural studies revealed direct interactions between yeast Lsm2 and Lsm3 subunits and the PatC domain (Sharif and Conti, 2013, Wu et al., 2014). Indeed, the PatC domain, and its interaction with Lsm2/3, is required for decapping in yeast and *Drosophila* (Braun et al., 2010, Wu et al., 2014).

Additional Pat1-binding proteins include DDX6 (Dhh1p), a DEAD box RNA helicase implicated in translational repression and decapping (reviewed in Ayache et al., 2015), and other decapping co-activators, including Edc3 and Edc4. Consistent with its role in mRNA decay, Pat1 also co-immunoprecipitates the CCR4-NOT deadenylase complex, Dcp1/2 decapping enzyme, and Xrn1 exonuclease (Braun et al., 2010, Haas et al., 2010, Ozgur et al., 2010).

Pat1 proteins and these interacting proteins are found concentrated in cytoplasmic foci, or processing bodies (P bodies), which are implicated in the control of mRNA storage and decay and contain translational repressors, decay enzymes, and mRNA (Decker and Parker, 2012, Eulalio et al., 2007). For example, in glucose-starved yeast, Pat1p is enriched in P bodies (Sheth and Parker, 2003, Teixeira and Parker, 2007), and human Pat1b is a component of P bodies present constitutively in mammalian cell lines and is required for their efficient formation (Ayache et al., 2015, Marnef et al., 2010, Ozgur et al., 2010).

At steady state, Pat1 proteins are predominantly cytoplasmic, but early clues in yeast that Pat1 could be nucleocytoplasmic proteins were more recently confirmed in mammalian cells (Marnef et al., 2012, Teixeira and Parker, 2007). Human Pat1b is confined to nuclei in the presence of the nuclear export inhibitor leptomycin B or when its nuclear export sequence (NES) is mutated, indicating that its export is mediated by Crm1 (Marnef et al., 2012) and hinting at a hitherto unidentified role of Pat1b in the nucleus. Here, we provide evidence for a nuclear Pat1b/Lsm2-8 complex enriched in Cajal bodies, in addition to the well-studied cytoplasmic Pat1b/Lsm1-7 complex in P bodies. This study considerably extends our previous observation of the nucleocytoplasmic shuttling nature of human Pat1b and identifies an unanticipated role of Pat1b in alternative splicing.

## Results

### The Pat1b Interactome Includes Predicted Cytoplasmic Partners and Tri-snRNP Components

**Figure 1:**
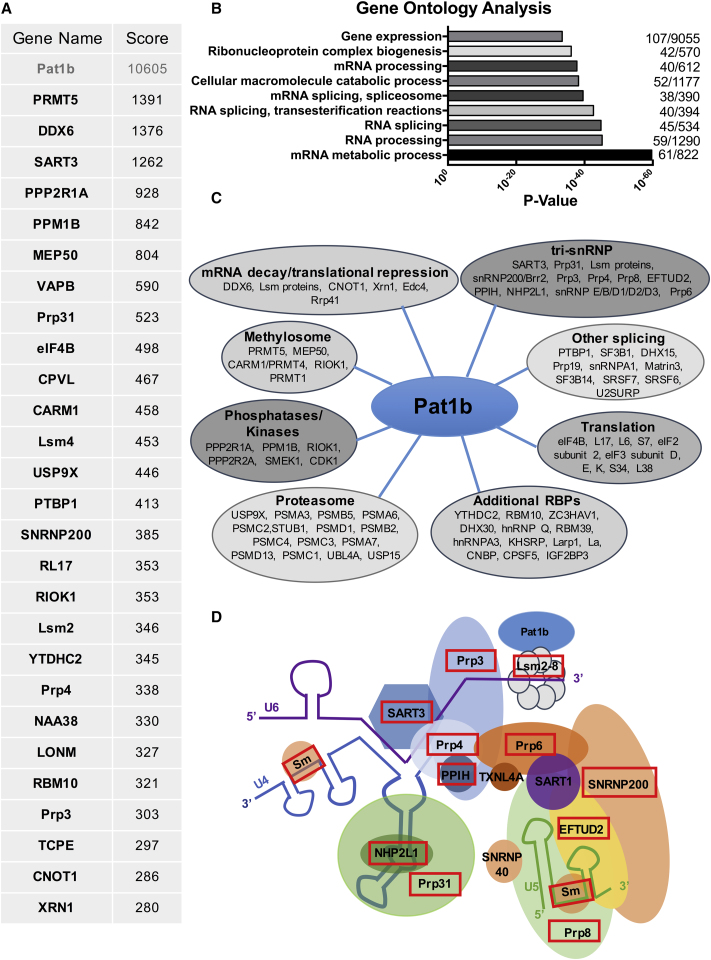
Pat1b Interactome Analysis (A) Mass spectrometry results of the top 27 proteins specifically co-immunoprecipitating with FLAG-Pat1b, ranked by MudPit score. (B) GO analysis of the high-confidence Pat1b-interacting proteins. (C) Schematic cartoon showing major complexes of Pat1b-interacting proteins. (D) Schematic cartoon of tri-snRNP protein and RNA constituents, adapted from Wahl et al. (2009). Peptides corresponding to boxed proteins co-immunoprecipitate with Pat1b. See also Tables S1 and S2.

The study was initiated by identifying Pat1b protein partners, based on the reasoning that these interacting factors could offer clues to its functions in the cytoplasm and nucleus. FLAG-Pat1b or FLAG-GFP control plasmids were expressed in HEK293 cells. The resulting total cell lysates were incubated with M2-Sepharose beads, and bound proteins were eluted with FLAG peptide and subsequently analyzed by mass spectrometry (Figure 1). A total of 166 proteins, represented by two or more unique peptides, interacted specifically with Pat1b, but not the control GFP. Figure 1A lists the top 27 interacting proteins, ranked by score (full details are given in Table S1). Gene Ontology (GO) analysis of these proteins revealed a highly significant association with RNA processing and splicing process terms (Figure 1B). Accordingly, many of the interacting factors could be classified into functional subgroups, including mRNA decay/translational repression, splicing, and RNA-binding protein categories, discussed further below (Figure 1C). Additional groups of co-factors included the methylosome, phosphatases/kinases, and proteasome subunits. The methylosome components are of interest as symmetric arginine methylation of Sm proteins and Lsm4 influence interactions with survival motor neuron protein (SMN) and P-body formation (Arribas-Layton et al., 2016, Brahms et al., 2001). Pat1b is a phosphoprotein, although the sites and consequences of phosphorylation are not fully characterized. In yeast, protein kinase A (PKA) phosphorylation of Pat1p disassembles P bodies and reduces long-term survival of stationary phase cells (Ramachandran et al., 2011), while serines 179 and 184 of human Pat1b are potential mTOR sites (Kang et al., 2013).

Focusing on the RNA-binding protein categories, FLAG-Pat1b immunoprecipitated its predicted cytoplasmic partners DDX6, Lsm proteins, CNOT1, Xrn1, and the decapping activator Edc4, reflecting its role in mRNA decay. Unexpectedly, the Pat1b immunoprecipitate also contained almost the entire set of tri-small nuclear ribonucleoprotein particle (tri-snRNP) factors, including SART3 (Prp24 in yeast), Prp31, snRNP200/Brr2, Prp4, and Prp3 (Figures 1A, 1C, and 1D; Table S1). The spliceosome assembles on pre-mRNA to define the boundaries between exons and introns using >150 proteins and U1, U2, U4, U5, and U6 snRNP complexes. Three of these join as a pre-formed unit called the tri-snRNP (U4/U6.U5), which recycles after each splicing event (reviewed Chen and Moore, 2014). SART3 (squamous cell carcinoma antigen recognized by T cells) promotes U4 and U6 small nuclear RNA (snRNA) annealing to form the U4/U6 di-snRNP, which then associates with U5 snRNP, mediated by Prp31 and Prp6 interactions to form the mature tri-snRNP. U6 snRNA uniquely interacts with Lsm2-8, rather than Sm proteins, that are common to U1, U2, U4, and U5 snRNP. The uridine-rich 3′ end of U6 snRNA binds the inner face of the Lsm2-8 heptamer (Zhou et al., 2014) and its telestem region, Prp24/SART3 (Montemayor et al., 2014). Prp3 and Prp4 interact with each other in U4/U6 di-snRNP, which also includes NHP2L1 and PPIH, while snRNP200, Prp8, and EFTUD2 are U5 snRNP proteins (Figure 1D; Agafonov et al., 2016, Bell et al., 2002, Nguyen et al., 2016, Wahl et al., 2009).

An alternative immunoprecipitation approach was used, based on GFP-Trap pull-down of YFP-Pat1b, also in HEK293 cells. Here too, we noted high retention of DDX6, CNOT1, Xrn1, Lsm proteins, and decapping factors, including Dcp1a, Dcp2, Edc4, and Edc3. Strikingly, DDX6 and SART3 were again among the top three interacting proteins (based on unique peptide number). In addition to SART3, YFP-Pat1b also bound Prp8, snRNP200, Prp3, Prp4, and NHP2L1 (Table S2).

Thus, two independent methods of purifying Pat1b-binding proteins demonstrated its known interactions not only with DDX6 and the 5′-3′ mRNA decay machinery but also, unexpectedly and specifically, with most of the tri-snRNP components.

### Verification of Pat1b/tri-snRNP Interactions

**Figure 2:**
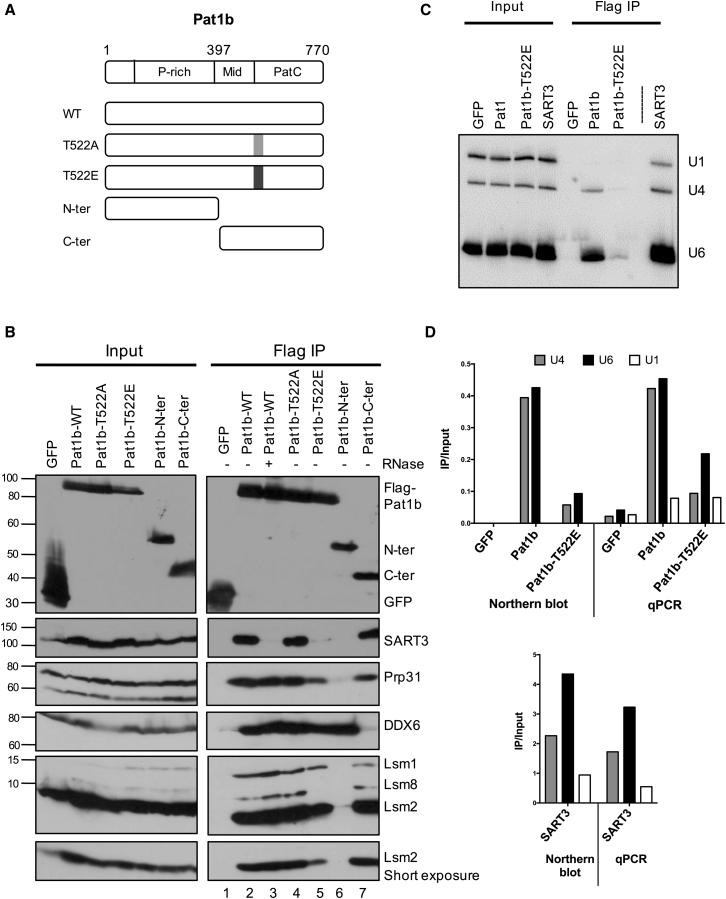
Pat1b Interacts with Tri-snRNP Protein and RNA Components (A) Schematic cartoon of FLAG-Pat1b constructs, including the N-terminal, C-terminal, and T552E/A constructs. (B) HEK293 cells were transiently transfected with FLAG-GFP and FLAG-Pat1b plasmids, and lysates were immunoprecipitated with M2 Sepharose. The full-length FLAG-Pat1b lysate was also treated with RNase A. Bound proteins were analyzed by western blotting with the indicated antibodies. (C) FLAG-tagged GFP, Pat1b, and SART3 immunoprecipitation was carried out as above. Northern blot analysis of RNA from bound fractions, with indicated snRNA probes. (D) Comparison of RNA bound to FLAG-tagged proteins determined by northern blot analysis and qRT-PCR, quantitated as ratio of immunoprecipitated (IP) RNA to input RNA. See also Figures S1–S4.

FLAG-Pat1b immunoprecipitation of selected protein and RNA tri-snRNP components was validated by western and northern blotting and qPCR (Figure 2). To identify regions of Pat1b necessary for co-purification, we tested Pat1b N-terminal and C-terminal constructs (Figure 2A). We also examined the effects of a Pat1b mutation, T522E, on protein and RNA binding. The mutation was hypothesized to impact Pat1b interactions on the basis of two criteria. First, the mutation introduces a negative charge in the basic patch formed by a set of ten arginine and lysine residues at the very N terminus of the PatC domain, shown to bind RNA (Braun et al., 2010). Second, T522 lies within the α1A helix, which binds Lsm3 and Lsm2, but it faces away from them (Figure S1; Sharif and Conti, 2013, Wu et al., 2014). In some experiments, we also used the charge-neutral T522A mutation.

DDX6 and Lsm1/2 interacted with full-length Pat1b efficiently and specifically, and this binding was not affected by RNase treatment, as shown previously (Braun et al., 2010, Ozgur et al., 2010). We also demonstrated an interaction with Lsm8, the specific component of the nuclear Lsm2-8 heptamer ring (Figure 2B, lanes 1–3). In line with prior studies, DDX6 binds the N-terminal and Lsm1/2, as well as Lsm8, the C-terminal region of Pat1b (Figure 2B, lanes 6 and 7).

As shown in Figure 2B, we next confirmed the efficient interaction of FLAG-Pat1b with SART3. We further verified that FLAG-SART3 immunoprecipitates Pat1b in a reciprocal pull-down experiment and provides evidence of the association between endogenous SART3 and Pat1b proteins (Figure S2). FLAG-Pat1b also interacted with Prp31, and both SART3 and Prp31 bind the C-terminal rather than N-terminal region of Pat1b (Figure 2B, lanes 6 and 7). However, the interaction between Pat1b and SART3 required both the Mid and PatC domains, whereas the PatC domain was sufficient for Prp31 binding to SART3 (Figure S3). Interestingly, efficient Lsm co-immunoprecipitation with Pat1b also required the two C-terminal domains (Braun et al., 2010). Moreover, while SART3 failed to bind Pat1b in the presence of RNase, Prp31 interacts with Pat1b in an RNA-independent manner (Figure 2B, lanes 2 and 3), together highlighting their distinct modes of binding.

We then examined the interaction of Pat1b with snRNA. FLAG-GFP, FLAG-Pat1b, and FLAG-SART3 transiently expressed proteins were immunopurified as described in Experimental Procedures, and retained RNA was extracted and submitted to northern blotting or qRT-PCR (Figures 2C and 2D). Confirming the presence of the tri-snRNP in Pat1b complexes, these approaches showed that Pat1b co-purifies with U4 and U6 snRNA, but not U1 snRNA, and it did so via its C-terminal region (Figures 2C, 2D, and S4). As expected, SART3 also preferentially co-immunoprecipitated U4 and U6 snRNA (Figures 2C and 2D).

The T522E, but not the T522A mutation, in Pat1b abolished its interaction with SART3, although neither affected DDX6 binding (Figure 2B, lanes 4 and 5). Similarly, the T-E mutation, but not the T-A mutation, reduced U4/U6 snRNA co-precipitation with Pat1b (Figures 2C, 2D, and S4). The effects of the T-E mutation seemingly mirrored RNase treatment of immunoprecipitated Pat1b (i.e., specific loss of SART3 binding as well as that of U4/U6 snRNA), suggesting a possible direct interaction between Pat1b and snRNA; however, we could not detect any interaction between recombinant Pat1bC-ter and U4/U6 snRNA in vitro (data not shown). Indeed, an alternative outcome of the mutation was indicated when monitoring Lsm interactions. As shown in Figure 2B, the T-E mutation disrupted Pat1b-Lsm8 binding, did not affect its interaction with Lsm1, and partly reduced its binding to Lsm2. The simplest interpretation of these observations is that the T-E mutation in Pat1b abrogates Lsm2-8 and thus also U6 snRNA/SART3 binding, but not the interactions with Lsm1-7.

Collectively, these data show that the C-terminal region of Pat1b binds to SART3, Prp31, Lsm proteins, and U4/U6 snRNA, with a different mode of interaction for SART3/snRNA versus Prp31 and for the nuclear versus cytoplasmic Lsm complex.

### Pat1b’s Interaction with SART3 and U6 snRNA Is Mediated by Lsm2-8

**Figure 3:**
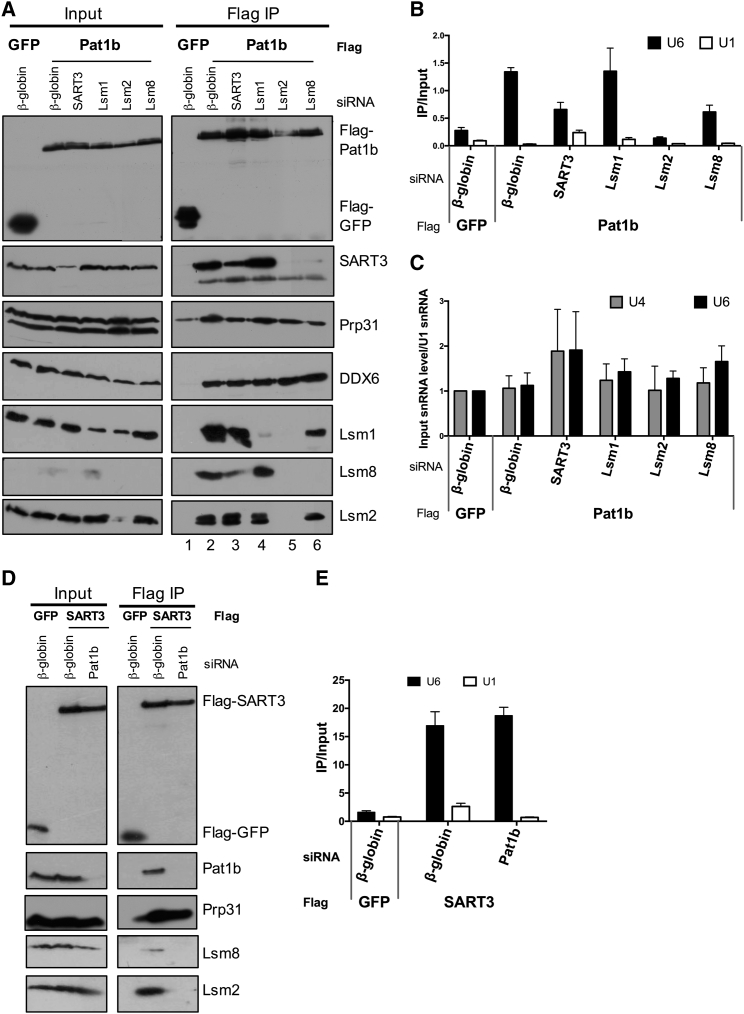
Interactions Linking Pat1b/Lsm2-8/U6 snRNA/SART3 (A) FLAG-GFP and FLAG-Pat1b plasmids were transiently transfected into HEK293 cells co-expressing control (β-globin) and specified siRNAs. Lysates were analyzed by western blotting as in Figure 2. (B) U6 and U1 snRNA bound by immunoprecipitated proteins was analyzed by qRT-PCR, as in Figure 2. Average and SD values for technical duplicates and error bars are shown. (C) snRNA levels are largely unaffected by SART3 or Lsm protein knockdown, as determined by qRT-PCR, using input samples from (B). U4/U6 snRNA levels were normalized to U1 snRNA. Average and SD values of three biological replicate experiments are shown. (D) FLAG-GFP and FLAG-SART3 plasmids were transfected into HEK293 cells along with control (β-globin) and Pat1b siRNAs. Lysates were analyzed by western blotting. (E) U6 and U1 snRNA bound by immunoprecipitated proteins was analyzed by qRT-PCR. Average and SD values for technical duplicates and error bars are shown. See also Figure S5.

To explore further the link between Pat1b and SART3, we used small interfering RNAs (siRNAs) to reduce SART3 and Lsm1/2/8 protein levels in cells co-expressing FLAG-GFP or FLAG-Pat1b plasmids and performed FLAG immunoprecipitation as before, followed by western blot and qRT-PCR analysis (Figure 3). Prp31 and DDX6 binding to Pat1b was unaffected by any depletion. Overall levels of SART3 were unaffected by siRNA treatments, although depleting Lsm2 unexpectedly reduced the levels of Lsm1 and Lsm8 as well as Lsm2, but such a co-reduction was not seen with Lsm1 or Lsm8 depletion (Figure 3, input lanes; also see Figure 6E). A similar effect was observed in HeLa cells (Figure S6). Strikingly, Pat1b co-precipitation of SART3 was prevented when Lsm2 or Lsm8 was depleted (Figure 3A, lanes 5 and 6). In contrast, reduced Lsm1 levels did not affect and may even enhance SART3 binding to Pat1b (Figure 3A, lane 4). In line with the idea that SART3/Pat1b binding is mediated by Lsm2/8, qRT-PCR of RNAs co-purifying with FLAG-Pat1b also showed Lsm2/8-specific effects. Lsm1 depletion did not significantly affect U6 snRNA binding by Pat1b, whereas SART3 and Lsm8 siRNA both reduced U6 snRNA binding, and Lsm2 completely abrogated it. Importantly, none of the depletions affected the abundance of U6 or U4 snRNAs (Figure 3C).

Pat1b thus co-immunoprecipitates SART3 via Lsm2, Lsm8, and U6 snRNA, linking Pat1b/Lsm2-8/U6 snRNA/SART3, as RNase treatment prevents this interaction (Figure 2), as do depletions of Lsm2 and Lsm8, but not Lsm1 (Figure 3). Moreover, the T522E mutation in Pat1b abrogates binding to Lsm8, SART3, and U6 snRNA, without altering the Lsm1 association (Figure 2).

These observations prompted us to examine the distribution and interactions of Pat1b and Lsm proteins in nuclei and the cytoplasm. Biochemical fractionation of HEK293 cells was monitored by western blotting using antibodies to ribosomal protein S6 and nuclear lamin A/C (Figure S5A). Endogenous Pat1b was present in both the cytoplasmic and nuclear fractions of untreated cells, consistent with our previous study (Marnef et al., 2012). We estimate that a significant pool of endogenous Pat1b (∼20%) at steady state is nuclear. Interestingly, Pat1b mobility was lower in the nucleus, suggesting that nuclear Pat1b is a modified (possibly phosphorylated) relative of the cytoplasmic form.

**Figure 4:**
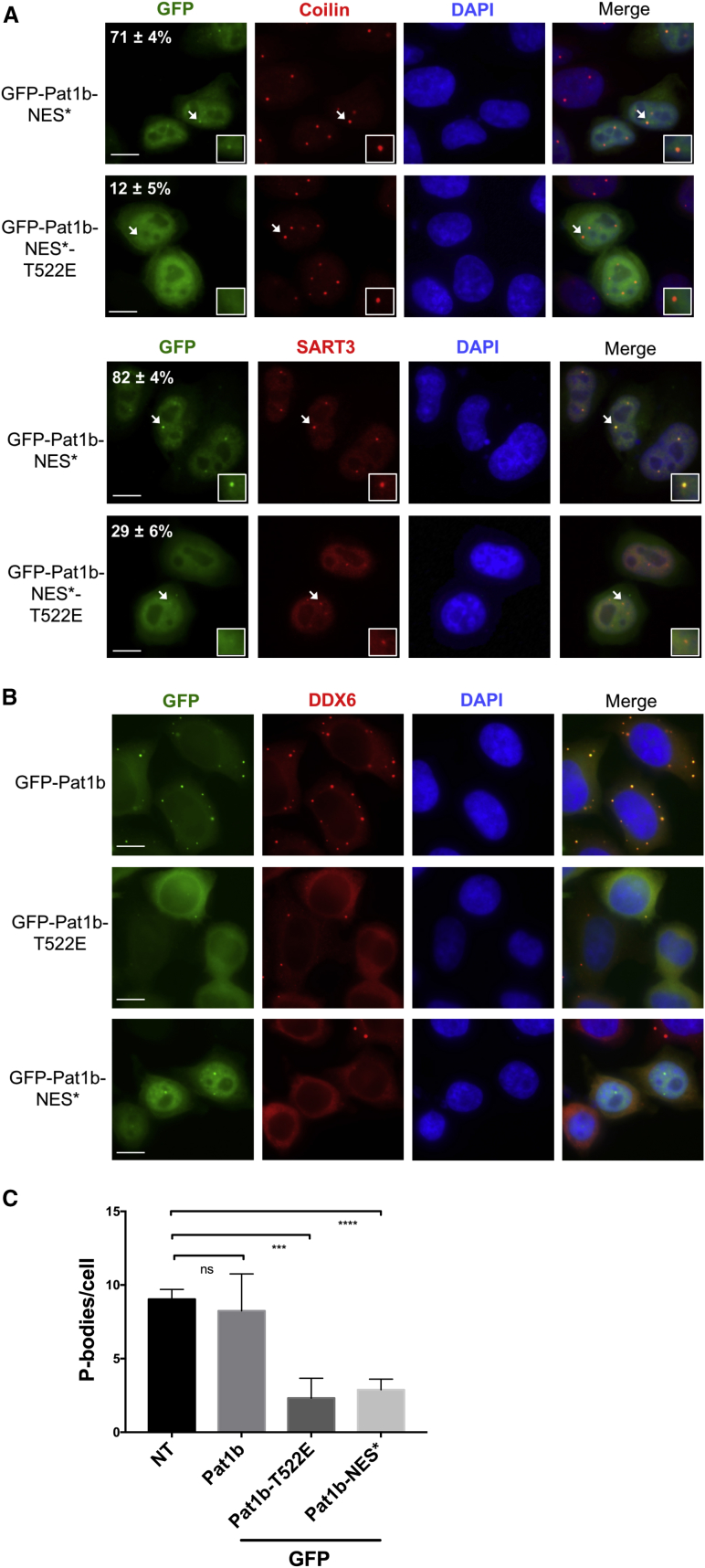
Pat1b Co-localizes with Coilin and SART3 in Cajal Bodies (A) HeLa cells were transiently transfected with GFP-Pat1b-NES plasmids, co-stained with SART3 or coilin antibodies (Cajal body markers), and processed for immunofluorescence. The frequency of Pat1b co-localization with Cajal bodies is indicated (in percentage: average and SD from three independent experiments). Scale bars, 10 μm. (B) HeLa cells were transfected with GFP-Pat1b plasmids, co-stained with DDX6 antibodies (P-body marker), and processed for immunofluorescence. DAPI staining was used to label nuclei. Scale bars, 10 μm. (C) Graph showing the number of endogenous P bodies per cell, detected by DDX6 antibodies, in cells expressing indicated GFP-tagged plasmids. Average and SD values from four independent experiments are shown. The significance was assessed by the two tailed t test (ns, p > 0.05; ^∗∗∗^p < 0.001; ^∗∗∗∗^p < 0.0001). See also Figure S6.

We then interrogated the effects of the Pat1b T522E mutation on Lsm interactions in nuclear and cytoplasmic fractions. Following transfection of HEK293 cells with FLAG-Pat1b or FLAG-GFP, lysates were fractionated into nuclear and cytoplasmic pools as above, and these were then subjected to FLAG antibody precipitation (Figure S5B). As expected, Lsm1 is cytoplasmic, while Lsm2 partitions across both fractions. Pat1b/Lsm1 binding was unaffected by the mutation, as in total lysates (Figure 2). However, the interaction with Lsm2 was preferentially affected by the nuclear mutant protein, rather than the cytoplasmic one (Figure S5B), explaining its partial reduction in total lysates (Figure 2). Of note, as shown by fractionation (Figure S5B) and immunofluorescence (Figure 4B), the T-E mutation does not alter the cytoplasmic/nuclear distribution of Pat1b.

Thus there are two compartmentalized Pat1b-Lsm complexes, the cytoplasmic Pat1b/Lsm1-7 and the nuclear Pat1b/Lsm2-8. Moreover, nuclear Pat1b, via Lsm2-8, interacts with U6 snRNA and SART3.

### Pat1b Stabilizes SART3-Lsm2/8 Interactions

To understand the possible role of Pat1b interactions with Lsm2-8 and SART3, we depleted Pat1b in cells transfected with FLAG-SART3 or FLAG-GFP. Following immunoprecipitation, western blotting showed loss of binding of Lsm2 and Lsm8 to SART3 when Pat1b levels were reduced, although SART3-Prp31 interactions were less affected (Figure 3D). In contrast, SART3-U6 snRNA binding was unaltered in Pat1b-depleted cells (Figure 3E). Co-precipitation of U6 snRNA by SART3 uncomplexed with Lsm2-8 is in line with in vitro data (Bell et al., 2002). Together, these data suggest that Pat1b enhances or stabilizes interactions between SART3 and Lsm2-8.

### Pat1b Co-localizes with SART3 and Coilin in Cajal Bodies

We next monitored Pat1b cellular distribution by immunofluorescence, first using the NES mutant form of GFP-Pat1b (Figure 4A; Marnef et al., 2012). In addition to diffuse nucleoplasmic localization, Pat1b-NES concentrated in small foci with coilin, one of the main Cajal body components, as well as with SART3, which is also enriched in Cajal bodies (Stanĕk et al., 2003). These non-membrane nuclear compartments are involved in aspects of short non-coding RNA metabolism, including snRNP biogenesis (reviewed in Staněk, 2016). Interestingly, the T-E mutation reduced Pat1b co-localization with coilin (from 71% to 12%) and SART3 (from 82% to 29%) (Figure 4A), in line with the severing of the co-immunoprecipitation with SART3 and U6 snRNA (Figures 2 and 3).

Wild-type Pat1b, whether endogenous or GFP tagged, is concentrated in cytoplasmic P bodies (Figure 4B; Braun et al., 2010, Marnef et al., 2010, Ozgur et al., 2010). Strikingly, the T-E mutation here not only prevented GFP-Pat1b from localizing to P-bodies but also was dominant negative for endogenous P bodies (Figures 4B and 4C), presumably reflecting changes in interacting protein association. Indeed, the NES form of Pat1b also reduces the number of P bodies per cell (Figure 4B, C), suggesting the possible co-redistribution of some of these factors to nuclei or abortive complexes formed by a key P-body factor trapped by the cytosolic mutant protein.

To further examine the possible interdependence of Cajal bodies and P bodies on Pat1b or Lsm proteins, we induced their knockdown by RNAi and used coilin and DDX6 antibodies to quantitate the number and size of the nuclear and cytoplasmic foci (Figures S6A and S6D). Cajal bodies were not significantly affected by Pat1b or Lsm1 depletion but were considerably enlarged both in size and number in the absence of Lsm2 or Lsm8 (Figure S6C). Such an increase may reflect the accumulation in Cajal bodies of di-snRNP stalled at the recycling step (Novotný et al., 2015, Schaffert et al., 2004). Pat1b, Lsm1, and Lsm2 depletion reduces P-body number, as reported previously for Pat1b and Lsm1 (see Introduction; Eulalio et al., 2007). In striking contrast, loss of Lsm8 very clearly enhances P bodies (Figure S6B). Indeed, as shown by Novotny et al. (2012), Lsm8 depletion results in nuclear Lsm4 being redistributed to the cytoplasm, leading to enhanced P-body formation. We conclude that the dynamic connection between these nuclear and cytoplasmic foci is particularly susceptible to the balance and distribution of nuclear and cytoplasmic Lsm proteins.

### Pat1b Depletion Stabilizes mRNAs Normally Found in P Bodies

**Figure 5:**
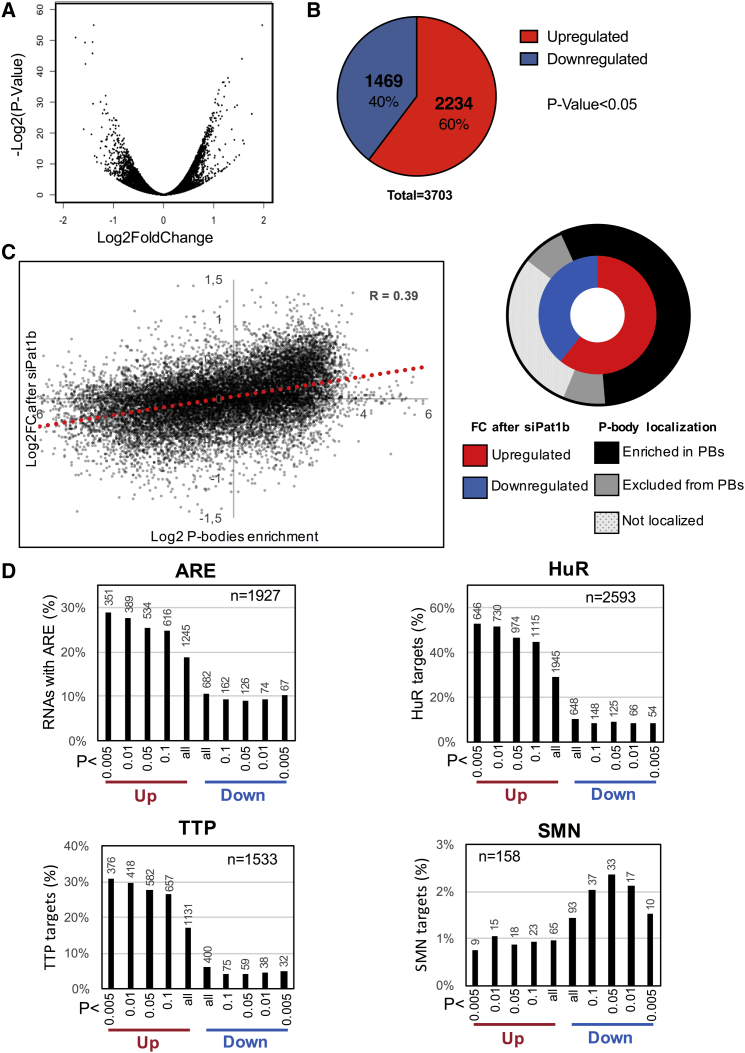
RNA-Seq Analysis of Transcriptome Changes following Pat1b Knockdown in HEK293 Cells (A) Volcano plot representing log2 fold changes in RNA expression and their associated p values. Differential gene expression was analyzed using DESeq2. (B) Pie chart showing the number of high-confidence (p value < 0.05) upregulated (in red, fold change > 0) or downregulated (in blue, fold change < 0) transcripts after Pat1b depletion. (C) Positive correlation between upregulation after Pat1b depletion and enrichment in P bodies. The dot plot represents the 16,451 RNAs common to both analyses, while the pie chart shows the strong overlap between transcripts significantly upregulated after Pat1b depletion (in red) and significantly enriched in P bodies (in black). (D) Enrichment of AU-rich element (ARE) mRNAs and targets of TTP and HuR in upregulated RNAs, and enrichment of SMN targets in downregulated RNAs. For each panel, the bars represent the percentage of transcripts of interest in the RNAs that are up- or downregulated after Pat1b depletion, while the tag above the bars is their absolute number. The total number of RNA targets in the Pat1b dataset is indicated. The enrichment is stronger for highly confident fold changes. See also Figure S7.

To assess the role of Pat1b in P-body- and snRNP-related processes, namely mRNA decay and splicing, the transcriptome of Pat1b and control-siRNA-treated HEK293 cells was analyzed by RNA sequencing (RNA-seq). Two biological replicates were analyzed, resulting in highly consistent data with pairwise Pearson correlations of >0.99 (Figures S7A and S7B). High-confidence changes (p < 0.05) in 3,703 transcript levels were observed, 60% of which were upregulated following Pat1b knockdown, in line with its role in mRNA decay (Figures 5A and 5B). GO analysis of this class revealed the enrichment of mRNAs involved in RNA metabolic processes and RNA-binding function, including small non-coding RNA and biogenesis, ribosome biogenesis, splicing, translation, mRNA decay, and other RNA-binding proteins. In contrast, the transcripts whose levels decreased upon Pat1b knockdown encoded proteins involved in developmental processes (Figures S7C and S7D).

Recently, P bodies were purified in HEK293 cells and their RNA content identified. A large variety of RNAs are specifically stored in P bodies, while others are excluded (A. Hubstenberger, M. Courel, M. Bénard, S., Souquère, M. Ernoult-Lange, R. Chouaib, Z. Yi, J.-B. Morlot, A. Munier, M. Fradet, M. Daunesse, M. Bertrand, G. Pierron, J. Mozziconacci, M. Kress, D.W., unpublished data). Interestingly, we found that RNA stabilization after Pat1b silencing correlated positively with enrichment in P bodies (correlation coefficient, 0.39) (Figure 5C). Indeed, 80% of the RNAs that were significantly stabilized were also significantly enriched in P bodies, compared to only 17% of the RNAs that were significantly downregulated (Figure 5C). Pat1b is a component of P bodies, and its depletion reduces their number (Figure S6; Ayache et al., 2015, Marnef et al., 2010, Ozgur et al., 2010). Hence Pat1b-dependent decay preferentially affects RNAs that are normally stored in P bodies.

Since AU-rich elements (AREs) are well-known determinants of mRNA degradation, we analyzed their frequency in Pat1b-sensitive mRNAs using the ARED database (Halees et al., 2008). ARE-containing mRNAs were much more frequent in upregulated mRNAs than in downregulated mRNAs, rising to 29% for the most significantly increased compared to less than 10% for downregulated ones (Figure 5D). The mRNA targets of two ARE-binding proteins, TTP and HuR, have been characterized by cross linking immunoprecipitation (CLIP) in HEK293 cells (Mukherjee et al., 2011, Mukherjee et al., 2014). Their link with Pat1b upregulation was even stronger than that of AREs. Up to 31% of the most significantly upregulated mRNAs were TTP targets, compared to less than 5% of the downregulated ones (Figure 5D). Similarly, up to 53% of the upregulated mRNAs were HuR targets, compared to less than 8% of the downregulated ones (Figure 5D). Thus, Pat1b-dependent decay preferentially affects ARE-containing mRNAs as well as TTP and HuR targets.

Searching for any factor related to downregulation after Pat1b silencing, we found that SMN-bound mRNAs (Rage et al., 2013) were twice more frequent in significantly down regulated mRNAs than in upregulated mRNAs (Figure 5D). This Pat1b-SMN link is probably even more significant, as SMN-bound mRNAs were characterized in the distant murine motor neuron-like NSC34 cell line. Although the exact function of SMN on these RNAs remains to be determined, it raises the possibility that Pat1b stabilizes, directly or indirectly, SMN targets.

### Pat1b Promotes Inclusion of Cassette Exons

**Figure 6:**
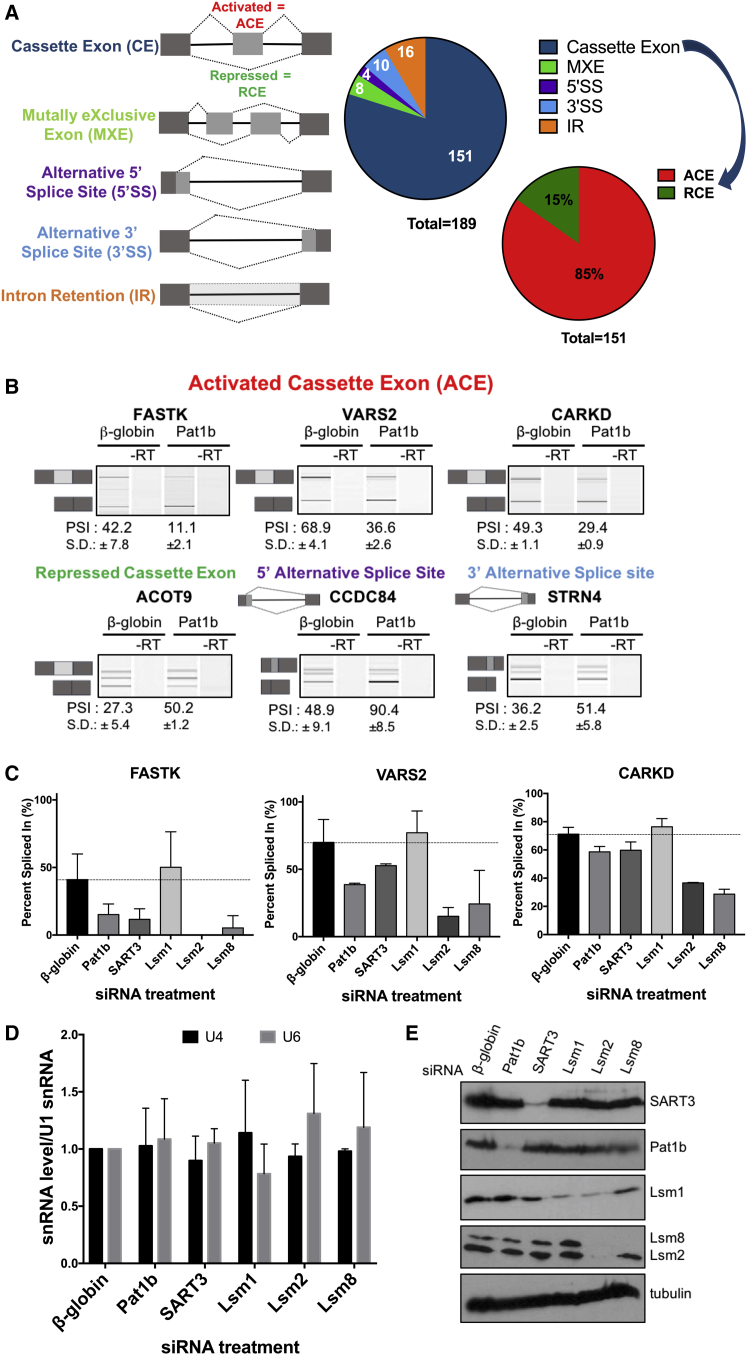
Splicing Regulation Mediated by Pat1b (A) Schematic representation of the different categories of alternative splicing and the summary of splicing changes following Pat1b depletion, revealed by RNA-seq analysis with rMATS pipeline. The first pie chart shows the number of events affected by Pat1b depletion in each category. Among regulated cassette exons, a second pie chart shows the distribution between activated and repressed CEs. (B) Validation of splicing events with RT-PCR. Values shown are mean ± SD of the PSI (percent spliced in) (n = 3). (C) HEK293 cells were treated with control (β-globin); Pat1b; SART3; Lsm1, 2, and 8 siRNA; and PSI for three ACE splicing events were assessed by RT-PCR. Mean ± SD values are shown (n = 3). (D) U4/U6 and U1 snRNA levels were determined by qRT-PCR. (E) Western blot verification of siRNA knockdown with indicated antibodies.

Next, we examined the potential role of Pat1b in regulating alternative splicing. Significant changes in splicing were predicted by rMATs. This identified 189 alternative splicing events (ASEs), with more than 80% at the level of inclusion or skipping of cassette exons (CEs). Of the regulated CEs, 85% showed decreased inclusion upon Pat1b depletion, suggesting that Pat1b normally promotes their inclusion (Figure 6A). We validated a number of these predicted ASEs, including examples of activated and repressed cassette exons (ACEs and RCEs), and alternative 3′ or 5′ splice sites. RT-PCR was performed using primers in flanking constitutive exons, and the percentage exon inclusion was determined (Figure 6B). These outcomes are unlikely to result from off-target effects of Pat1b RNAi, as similar results were noted for the three ACE transcripts in cells depleted of SART3, or Lsm 2 or Lsm8, with Lsm2/8 knockdowns showing the greatest effects. Significantly, Lsm1 depletion did not influence their alternative splicing, and none of the depletions changed snRNA levels (Figure 6C-E).

**Figure 7:**
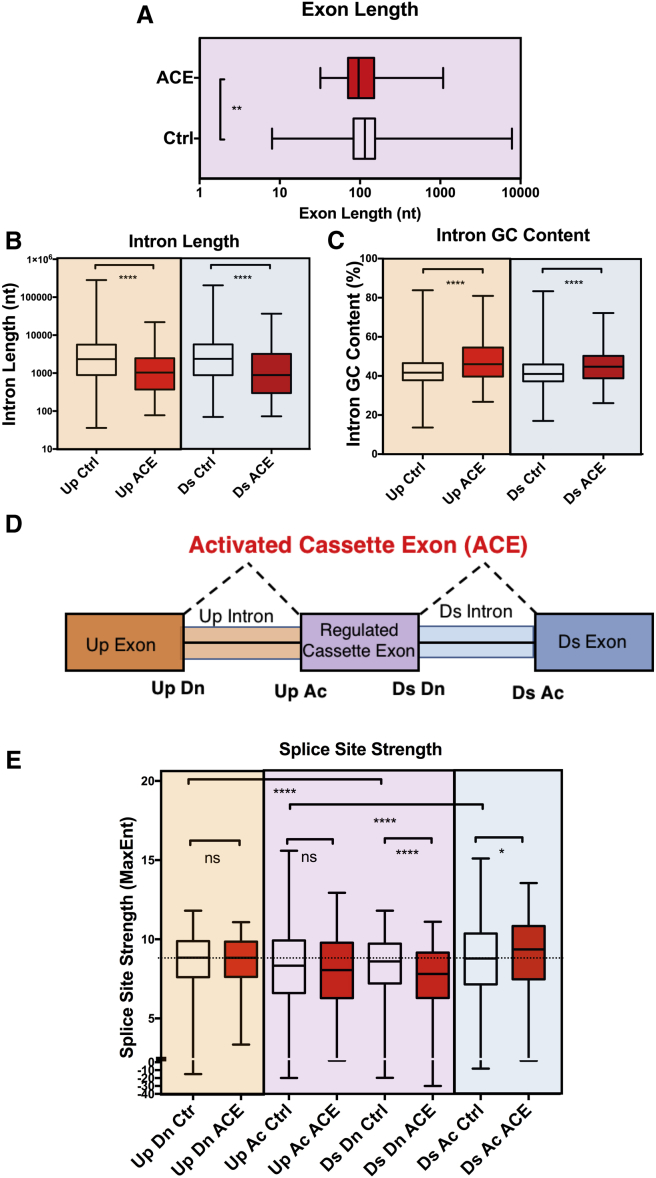
Properties of Pat1b-Regulated Cassette Exons (A) Length distribution of Pat1b-regulated exons. (B) Length distribution of introns (upstream and downstream). (C) Intron GC content (percentage). (D) Schematic cartoon of features of activated cassette exons regulated by Pat1b. Up, upstream; Ds, downstream; Dn, donor; Ac, acceptor. (E) Splice site strength (maximum entropy). A two-sided Mann-Whitney test was used to compare properties between datasets (ns, p > 0.05; ^∗^p < 0.05; ^∗∗^p < 0.01; ^∗∗∗^p < 0.001; ^∗∗∗∗^p < 0.0001).

To assess whether the exons regulated by Pat1b possess any specific splicing features they were compared to a control set of 16228 CEs that were insensitive to Pat1b depletion (Figure 7). While ACEs tended to be modestly but significantly shorter than control exons, their flanking introns were strikingly shorter (by a median of 1,030 nt compared to 2,337 nt for the control), with higher GC content (by a median of 46% versus 41% for the control for upstream intron and 44% versus 41% for the downstream intron) (Figures 7A–7C). We then analyzed the strength of the different splice sites. As expected, the regulated cassette exons have weaker donor and acceptor splice sites compared to the constitutive exons in the control set. The 5′ splice sites (Figure 7D, Ds Dn) of the ACEs were significantly weaker than those of the control CEs, whereas the 3′ splice site of the constitutive exon (Ds Ac) were slightly but significantly stronger (Figure 7E). Altogether, these data indicate that Pat1b enhances the inclusion of cassette exons that have significantly weaker donor and acceptor sites than their flanking constitutive exons and that are flanked by short introns.

## Discussion

### Two Pat1-Lsm Complexes

This study reports that Pat1b, in addition to its well-known interactions with the cytoplasmic Lsm1-7 heptamer, also associates with its nuclear Lsm2-8 counterpart. The X-ray crystal structure of the yeast Pat1C /Lsm1-7 complex was recently solved and shown to be bridged by Lsm2 and Lsm3 interactions (Sharif and Conti, 2013, Wu et al., 2014), consistent with a nuclear Pat1b/Lsm2-8 complex. Indeed, modeling of the yeast Lsm2-8 heptamer (Zhou et al., 2014) onto the PatC domain (Sharif and Conti, 2013) revealed considerable overlap between the two rings and compatibility of PatC with Lsm2-8 interactions (Figure S1). The nuclear Pat1/Lsm complex is likely to be conserved, as *Drosophila* HPat can accumulate in nuclei (Pradhan et al., 2012), and fungal Pat1 shuttles through nuclei in *Saccharomyces cerevisia* (*S. c.*) (Teixeira and Parker, 2007) and *Schizosaccharomyces pombe* (*S. p.*) (Wang et al., 2017).

Interestingly, changing Pat1b T522 to glutamic acid differentially impacts its binding to Lsm8 and Lsm1. This mutation, located close to the Lsm2/3-Pat1b interface, introduces a possible phosphomimetic residue in a predicted PKA consensus site, KRRKT^522^LVI, which lies in a very similar location to the yeast Pat1p PKA site, RRRS^456^S^457^Y (Ramachandran et al., 2011). Similarly to the outcome of this phosphorylation in yeast Pat1p, the T522E mutation was dominant negative for endogenous P bodies. Also of note, nuclear Pat1b appears to be modified relative to its cytoplasmic form. We speculate that the difference of Pat1b/Lsm complexes in the cytoplasm and nuclei, whether due to different Pat1b conformation or distinct co-factors, possibly results from differential phosphorylation.

How Pat1b binds RNA is also of interest in regard to how it functions, particularly as it lacks any known RNA-binding domains or motifs. Pat1 proteins have been shown to bind mRNA via the Lsm1-7 complex as well as in an Lsm-independent manner (Braun et al., 2010, Chowdhury et al., 2014, Chowdhury et al., 2007, Haas et al., 2010, Mitchell et al., 2013, Pilkington and Parker, 2008). However, detailed understanding of these interactions is still lacking. This study, which shows that Pat1b can also bind Lsm2-8 and in turn U6 snRNA, widens the scope for future investigations of Pat1-RNA interactions.

### The Pat1b/Tri-snRNP Connection

The extended interactions linking Pat1b/Lsm2-8 to U6 snRNA and in turn SART3 were demonstrated using mass spectrometry, immunoprecipitation coupled with northern and western blotting, and RNAi depletion. The well-established binding of Lsm2-8 to U6 snRNA as well as now to Pat1b is compatible with structural studies. The U-rich 3′ end of U6 snRNA binds the inner core of the heptamer ring (Zhou et al., 2014), with the PatC domain interacting with the outward-facing residues of Lsm2-3 subunits (Figure S1; Sharif and Conti, 2013, Wu et al., 2014). Furthermore, Prp24/SART3 binds the telestem region of U6 snRNA and Lsm2-8 to its 3′ end (Montemayor et al., 2014). Altogether, these observations suggest that Pat1b participates in some aspect of tri-snRNP biogenesis, as this is the well-established role of SART3, which re-anneals U4 and U6 snRNA, and of Lsm2-8 (Bell et al., 2002, Verdone et al., 2004). The interaction of Pat1b with SART3 and U5 proteins suggests that Pat1b may interact with all snRNPs containing the Lsm2-8 ring (U6, U4/U6, and U4/U6.U5 snRNPs).

We also documented the specific additional binding of tri-snRNP proteins to Pat1b, albeit with a mode of binding distinct from SART3. In the case of Prp31, its binding to Pat1b was RNA and Lsm independent and only mediated by the PatC domain. The other tri-snRNP proteins could not be explored in any great detail, largely owing to lack of antibodies. However, a recent proteomic analysis of column-based fractionations of nuclear and cytoplasmic HeLa and HEK293 cell proteins identified several hundred soluble complexes (Havugimana et al., 2012). Interestingly, one of the largest complexes (618) includes Pat1b and tri-snRNP components (Prp31, Prp3, Prp4, Prp6, Prp8, and Sm) and additional splicing proteins, but not SART3, which is consistent with the nuclease treatment used to solubilize protein complexes (Havugimana et al., 2012). Interestingly too, yeast Pat1p was previously detected in U6-containing penta-snRNP (Stevens et al., 2002).

### Dual RNA-Processing Functions of Pat1b

High-throughput RNA-seq following Pat1b silencing showed changes in both transcript levels and alternative splicing, which affected different sets of genes. Pat1 proteins may have additional roles in regulating gene expression. In *S. cerevisiae*, with relatively low levels of splicing and alternative splicing (AS) restricted to regulated intron retention (Pleiss et al., 2007), deletion of Lsm2-5 and 8 or Pat1p results in delayed rRNA processing (Kufel et al., 2003, Muppavarapu et al., 2016). Our RNA-seq data lack information on rRNA species, as the RNA was subjected to RiboZero treatment. Nevertheless, our mass spectrometry data, documenting multiple interactions with mRNA decay machinery and tri-snRNP components, do not support a major role for human Pat1b in rRNA processing.

The mRNAs normally destabilized by Pat1b include many with functions in RNA metabolism and RNA binding, in line with idea that RNA binding proteins (RBPs) are part of cross-regulatory post-transcriptional networks, the “regulator-of-regulator” concept (reviewed Iadevaia and Gerber, 2015). Less anticipated was our observation that the upregulated transcripts showed a significant correlation with mRNAs purified from P bodies in untreated cells and with ARE mRNAs. Taking into account the previous model that P bodies could sequester untranslated ARE-containing mRNAs upstream of their decay (Franks and Lykke-Andersen, 2007), these data altogether suggest that Pat1b is specifically involved in this decay pathway.

We noted a relatively modest number of AS changes when Pat1b was depleted, possibly reflecting the minor proportion of nuclear Pat1b in proliferating HEK293 cells. As judged by the low frequency of intron retention events, Pat1b knockdown does not affect global splicing efficiency. Rather, Pat1b tends to enhance the inclusion of shorter than usual exons, with particularly weak splice sites compared to their adjacent constitutive exons. Weaker splice sites are usually thought to affect initial recruitment of early splicing factors, including U2AF, U1, and U2 snRNPs. We suggest that Pat1b normally enhances a step in tri-snRNP assembly by stabilizing SART3/Lsm2-8 interactions, without affecting snRNA levels. When tri-snRNPs are abundant, those exons are included, despite their weaker splice sites, possibly because the weak binding of earlier factors is rapidly stabilized by subsequent binding of tri-snRNP. However, when Pat1b is silenced, tri-snRNP is reduced and its inclusion is less efficient. Consistent with this possibility, we note that Pat1b-regulated exons are flanked by considerably shorter than usual introns, which would be transcribed faster than normal, leading to reduced recognition of weak splice sites when tri-snRNP levels are suboptimal. Indeed, cassette exons sensitive to the rate of transcription elongation have shorter flanking introns and weaker splice sites (Fong et al., 2014). It could seem paradoxical that a decrease in tri-snRNPs, which are required for all splicing events, only affects AS. However, a high-throughput siRNA screen of core and auxiliary regulatory splicing factors, including a number of tri-snRNP components, revealed that their depletion, rather than resulting in a general uniform inhibition of splicing, induced differential effects upon >35 functionally important AS events (Papasaikas et al., 2015). We propose therefore that Pat1b, which by virtue of its interactions with tri-snRNP likely acts late in the splicing complex assembly, can nevertheless affect splice site choice. Further support for this model comes from a recent kinetic study that showed that all steps in spliceosome assembly up to and including U4/6.U5 tri-snRNP recruitment are reversible and thus potential points of regulation (Hoskins et al., 2016).

In summary, this work identifies the dual pre/mRNA processing roles of Pat1b, an RNA-binding protein that is mostly cytoplasmic in mammalian tissue culture cells but shuttles in and out of nuclei and is a component of both P bodies and Cajal bodies. We speculate that the cellular distribution of Pat1b may differ in other cell types, likely altering the balance of its effects on gene expression.

## Experimental Procedures

Additional methods are detailed in Supplemental Experimental Procedures.

### Mass Spectrometry

HEK293 cells were transiently transfected with either FLAG-Pat1b or FLAG-GFP plasmids 24 hr after plating. After a further 24 hr, cells were harvested and the tagged proteins purified with anti-M2 affinity gel (Sigma). Bound proteins were eluted using FLAG peptide (Sigma) and separated briefly by SDS-PAGE, as previously described for FLAG-4E-T (Kamenska et al., 2016). Each lane was sliced into four slices, and tryptic peptides obtained by in-gel digestion were identified using one-dimesional sodium dodecyl sulfate-polyacrylamide gel electrophoresis followed by liquid chromatography-tandem mass spectrometry (GELC/MS/MS) in the Cambridge Centre for Proteomics. Peptide analysis was carried out with Proteome Discoverer using the SwissProt database.

### Immunoprecipitation, Western Blot Analysis, and qRT-PCR

Immunoprecipitation of FLAG-tagged proteins was performed with lysates from transfected cells incubated with M2 beads (Sigma Aldrich), and after washing, bound proteins and RNA were eluted and analyzed by western blotting or qRT-PCR (see Supplemental Experimental Procedures).

### HeLa Cell Culture, Transfection, and Immunofluorescence

HeLa cells were maintained in DMEM supplemented with 10% fetal bovine serum. Transient transfections were performed as described above with cells plated on 13-mm glass coverslips in 24-well plates. Cells were fixed in 4% paraformaldehyde, washed with PBS, and permeabilized in PBS with 0.5% Triton X-100 followed by PBS washes. Cells were incubated with primary antibodies for 1 hr. The cells were washed in PBS, followed by the incubation of the secondary antibodies conjugated to rhodamine or Alexa 488 used at a 1:1,000 dilution (Jackson ImmunoResearch Laboratories) for 1 hr. After rinsing three times with PBS, cells were stained with DAPI. The coverslips were mounted in Citifluor medium (Citifluor). All steps were performed at room temperature. Cells were observed under a Zeiss Axioimager M1 fluorescence microscope.

### RNA-Seq

RNA from two biological replicates each of β-globin or Pat1b siRNA-treated HEK293 cells was extracted with TriReagent and analyzed in the DNA sequencing facility of the Department of Biochemistry (University of Cambridge). Ribo-Zero TruSeq stranded mRNA libraries were prepared for each sample and sequenced on Illumina NextSeq 500 Sequencing System providing around 100 million reads per sample (∼75-bp paired-end reads). See Supplemental Experimental Procedures for details regarding RNA-seq analysis.

### Statistical Methods

The number of P bodies per cell and co-localization of GFP-Pat1b proteins with coilin/SART3 in Cajal bodies were quantitated in ∼50 cells in three or four independent experiments, respectively, using ImageJ. The significance was assayed by two-tailed t test (not significant [ns], p > 0.05; ^∗∗∗^p < 0.001; ^∗∗∗∗^p < 0.0001).

The two-sided Mann-Whitney test was used to compare properties between regulated cassette exon datasets (ns, p > 0.05; ^∗^p < 0.05; ^∗∗^p < 0.01; ^∗∗∗^p < 0.001; ^∗∗∗∗^p < 0.0001). All statistical analyses were performed with RStudio.

## Author Contributions

C.V. performed most of the experiments, with A.M., H.B., L.T., S.O., and G.S. also contributing. M.L., C.W.S., and J.M. helped with bioinformatic analysis. D.W. performed the analyses in Figures 5C and 5D. N.S. conceived the study and drafted the paper, which was edited by C.V., A.M., L.T., G.S., C.W.S., and D.W.

## Accession Numbers

The accession number for the RNA-seq data reported in this paper is ArrayExpress: E-MTAB-5577.

## Supplemental Information

Document S1. Supplemental Experimental Procedures and Figures S1–S7

Table S1. Mass Spectrometry Results of Proteins Co-immunoprecipitating with FLAG-GFP, Related to Figure 1 Only proteins with two or more peptides, and none in the control FLAG-GFP samples, are shown. Proteins are ranked by MudPit Score.

Table S2. Mass Spectrometry Results of Proteins Co-immunoprecipitating with YFP-Pat1b and YFP Alone, Ranked by Unique Peptide Number, Related to Figure 1 In bold are proteins also found in Table S1, and in italic those previously identified as Pat1b-interacting factors.

Document S2. Article plus Supplemental Information
