# Supplementary material for: Dual RNA Processing Roles of Pat1b via Cytoplasmic Lsm1-7 and Nuclear Lsm2-8 Complexes
Source: Cell Rep. 2017 Aug 1;20(5):1187–200. doi: 10.1016/j.celrep.2017.06.091 (PMC5554784; doi:10.1016/j.celrep.2017.06.091)
Supplement: Document S1. Supplemental Experimental Procedures and Figures S1–S7 [file mmc1.pdf]

Cell Reports, Volume *20*

Supplemental Information

Dual RNA Processing Roles of Pat1b via Cytoplasmic Lsm1-7 and Nuclear Lsm2-8 Complexes

Caroline Vindry, Aline Marnef, Helen Broomhead, Laure Twyffels, Sevim Ozgur, Georg Stoecklin, Miriam Llorian, Christopher W. Smith, Juan Mata, Dominique Weil, and Nancy Standart

Supplemental Figures

Figure S1

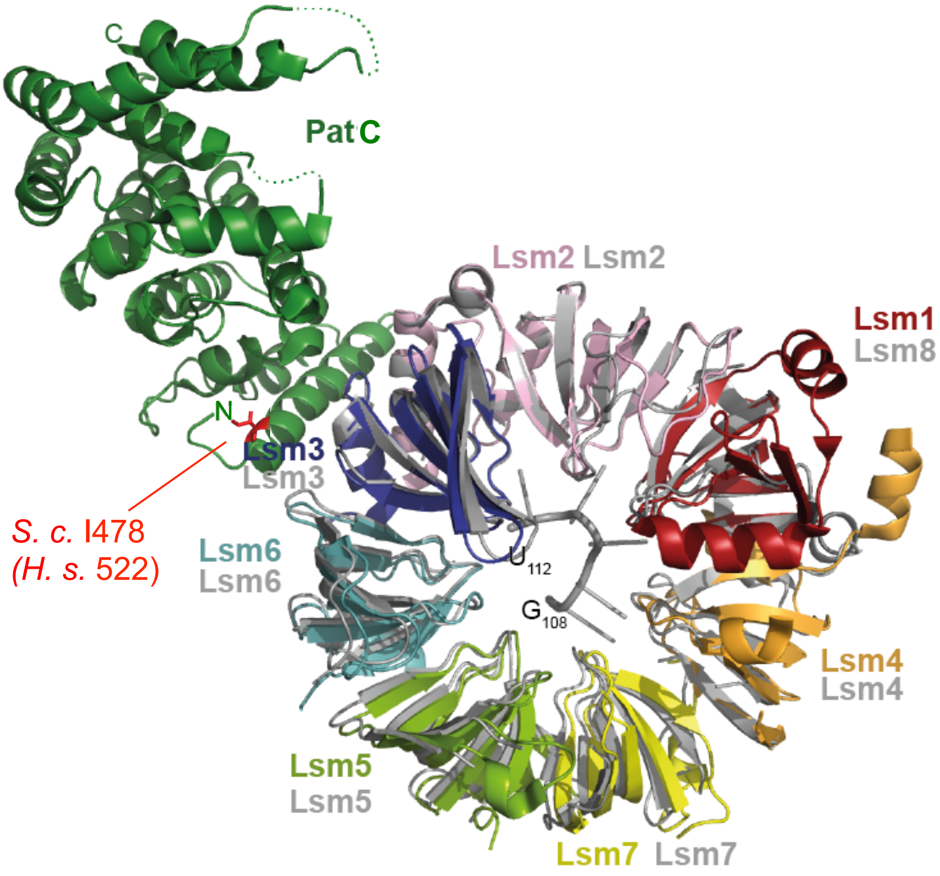

Figure S1. Superposition of the crystal structures of *S. cerevisiae* Lsm1$_B$-7-Pat1$_C$ and Lsm2-8 complex bound to 3' end of U6 snRNA. Related to Figure 2.
Superposition of the crystal structures of *S. cerevisiae* Lsm1$_B$-7-Pat1$_C$ (PDB: 4C8Q) (Sharif and Conti, 2013) and Lsm2-8 complex bound to 3' end sequence of U6 snRNA (PDB: 4M7A) (Zhou et al., 2014), viewed on the distal face. The Lsm1$_B$-7-Pat1$_C$ complex is coloured as in (Sharif and Conti, 2013), and the Lsm2-8 complex and the 5 nucleotides of the U6 snRNA are shown in gray. Other than minor changes both the Lsm rings align very well, indicating that Pat1 or RNA binding doesn't change their conformation. Also indicated in red is the position of the yeast equivalent of human Pat1b T522, I478, which is facing away from the Lsm1-7 ring, although very close to Lsm3 (Pat1 L479 interacts with Lsm3 L10).

Figure S2

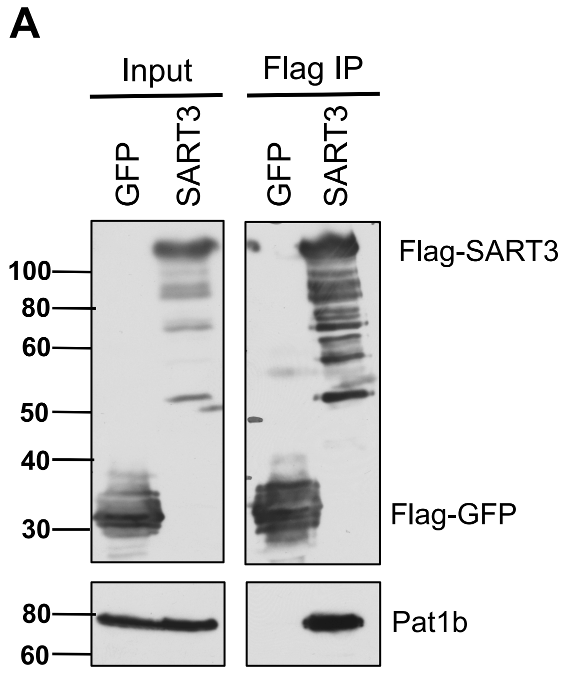

Figure S2. SART3-Pat1b binding examined using FLAG-SART3 and antibodies to endogenous proteins. Related to Figure 2.

A. FLAG-GFP and -SART3 immunoprecipitations were carried out as in Figure 2, and analysed by Western blotting with indicated antibodies. **B.** HEK293 cell lysate was immunoprecipitated with antibodies against Pat1b and SART3 and analysed by Western blotting.

Figure S3

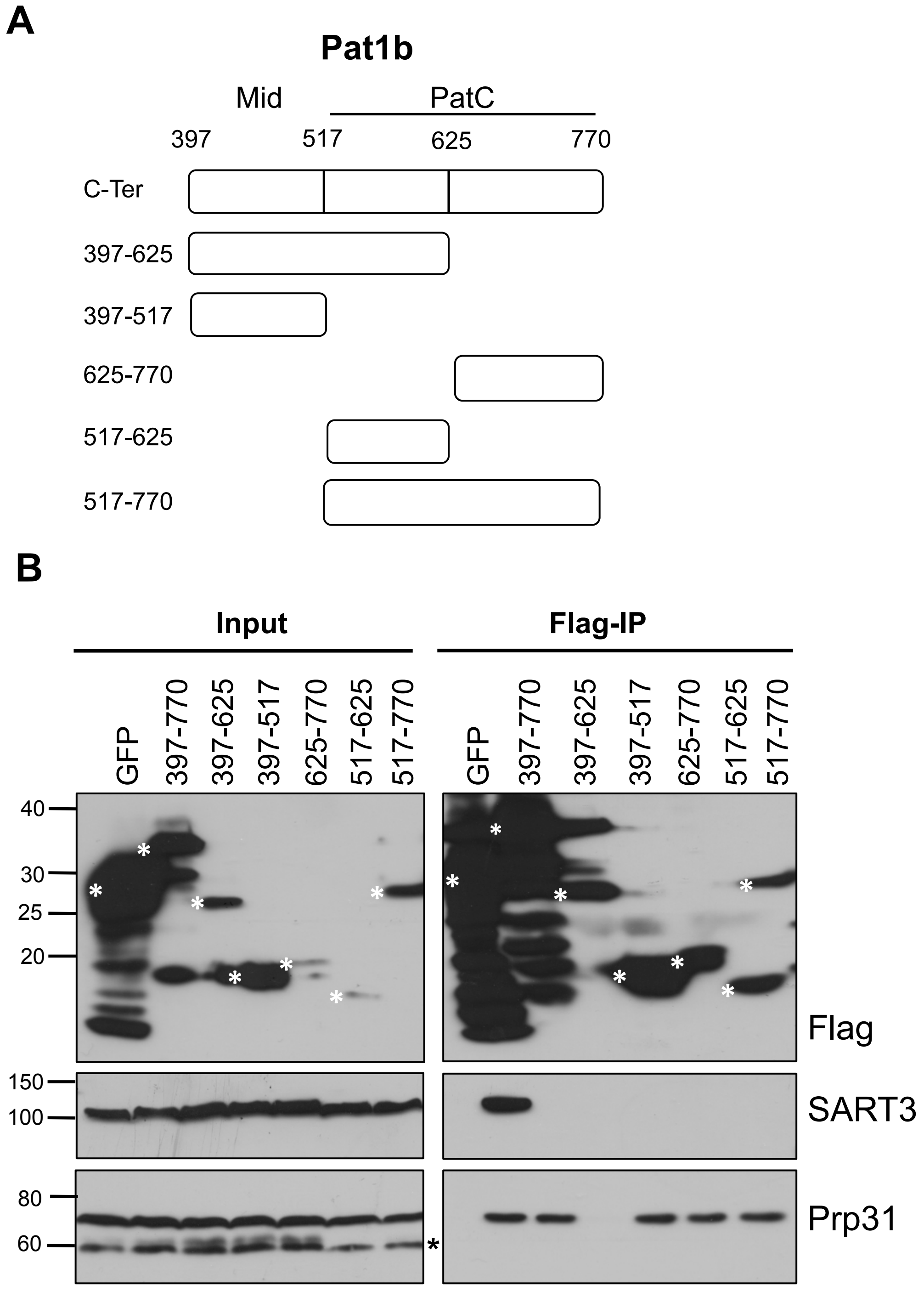

Figure S3. SART3 binding to Pat1b requires both Mid and PatC domains, while Prp31 only PatC. Related to Figure 2.

A. Schematic cartoon of Flag-Pat1bC-ter constructs. **B**. FLAG-GFP and –Pat1b-Cter, full-length and truncated versions as shown, immunoprecipitations were analysed by Western blotting. Black * indicates unrelated band. White * indicates Flag-Pat1b bands of expected size.

Figure S4

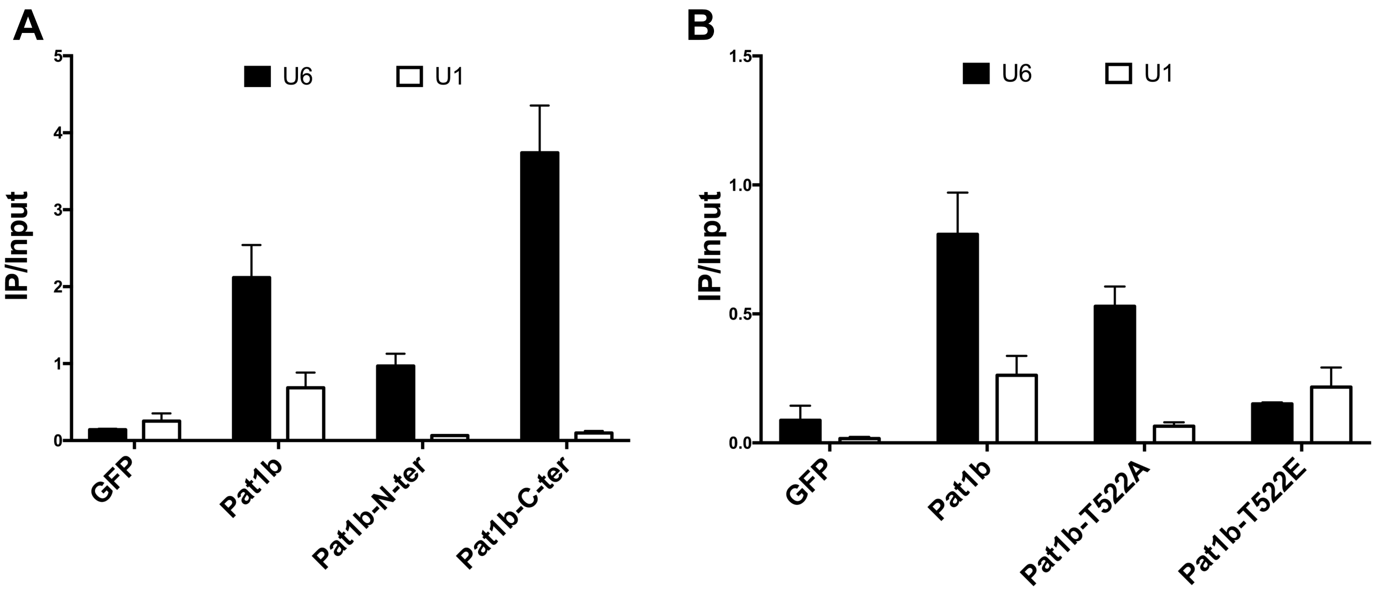

Figure S4. Pat1b-snRNA interactions analysed by qRT-PCR. **Related to Figure 2.**
A. Pat1b C-ter interacts with U6 snRNA. **B.** Pat1b binding to U6 snRNA is sensitive to T522E mutation**.** Average values and Error bars refer to three technical replicates in a representative experiment (n=3).

Figure S5

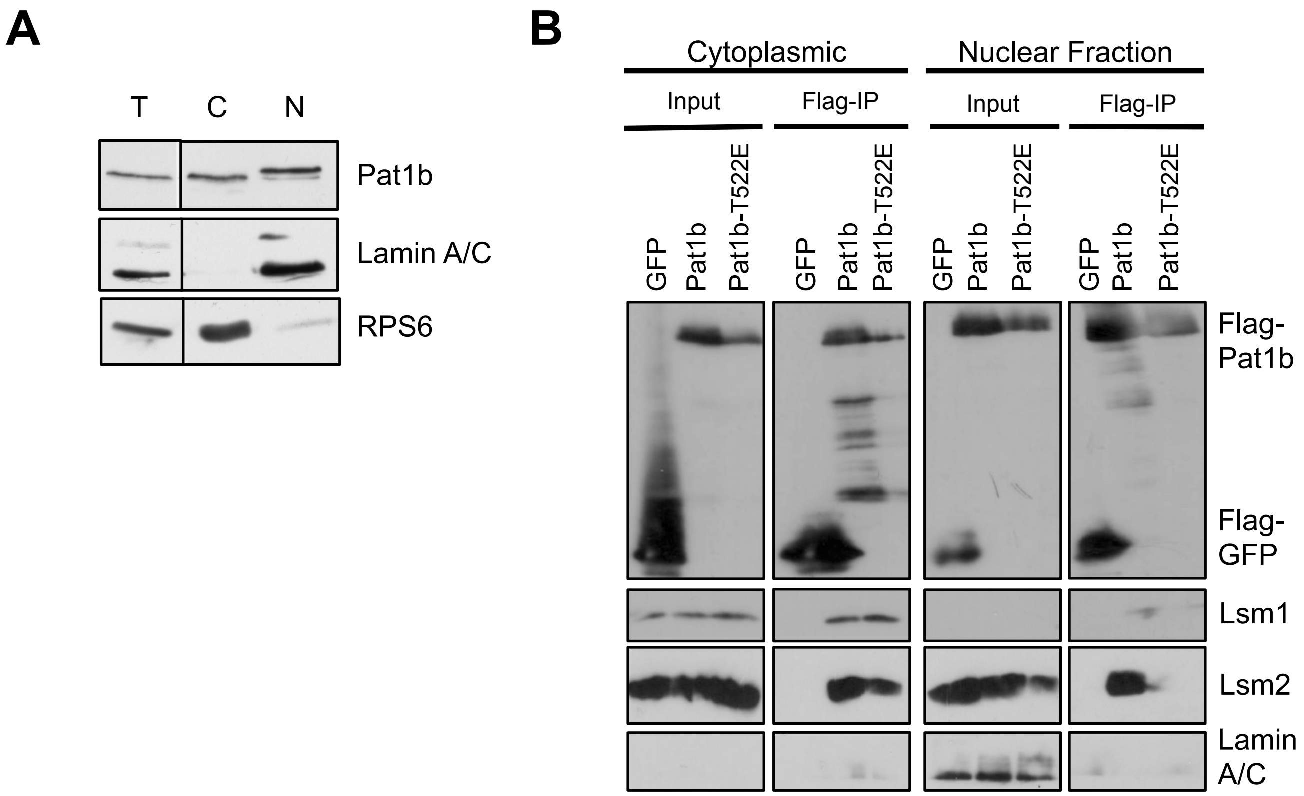

Figure S5. Two compartmentalized Pat1b-Lsm complexes. Related to figure 3.
A. HEK293 cells were separated into nuclear and cytoplasmic fractions. Western blotting analysis was performed with 15% or 20% SDS-PAGE with equal amounts of cytoplasmic and nuclear proteins, representing 2 % and 8 % of their starting amounts. **B**. HEK293 cells were transiently transfected with FLAG-GFP and -Pat1b plasmids, and separated into nuclear and cytoplasmic fractions, used in immunoprecipitation assays, as in Figure 2. Western blotting analysis was performed using the same ratio of fractions as described in **A**.

Figure S6

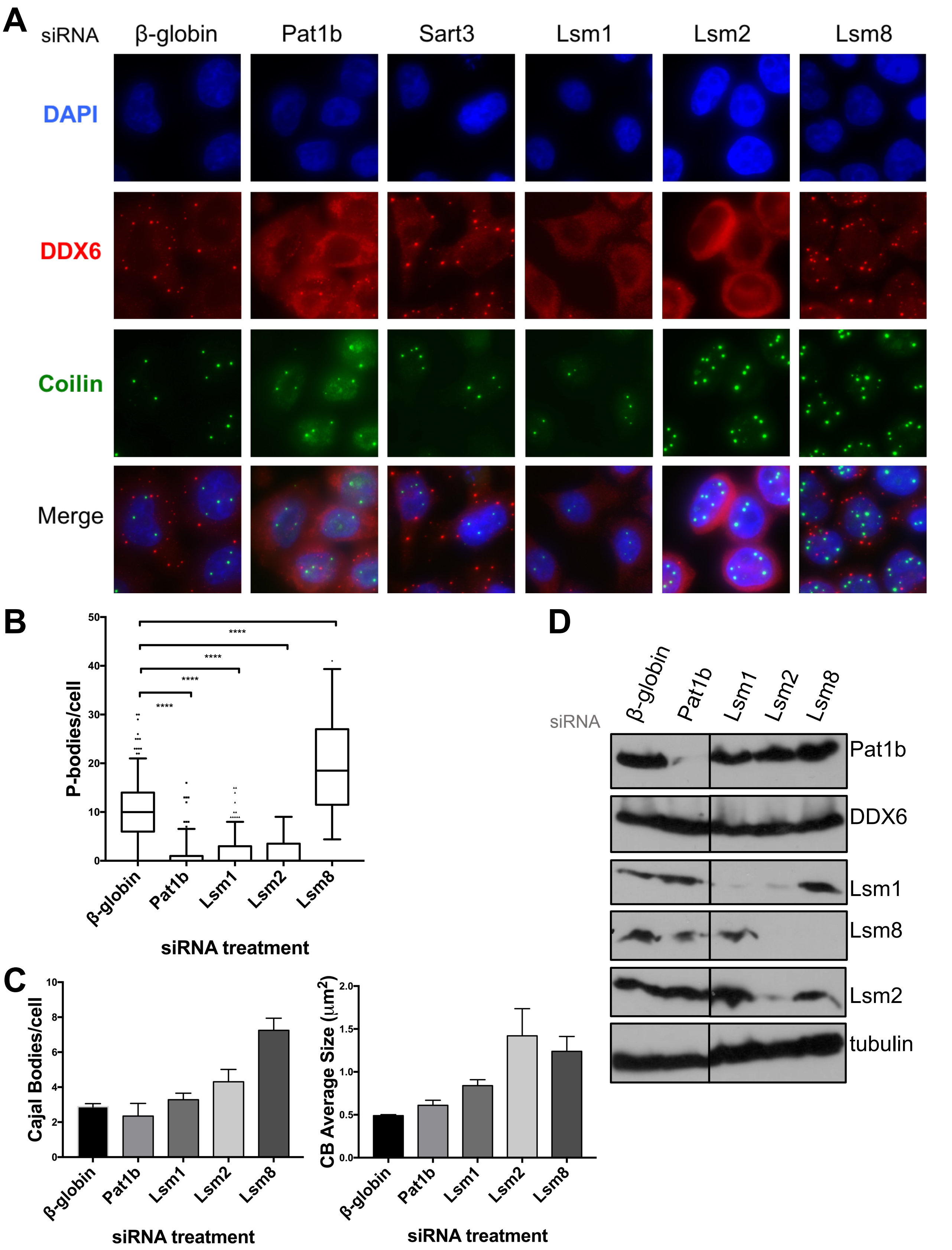

Figure S6. Distinct effects of Pat1b-Lsm protein knock-down on P-bodies and Cajal bodies. Related to Figure 4.

A. Hela cells were treated with control (β-globin), Pat1b and Lsm1, 2 and 8 siRNA, and cells were processed for immunofluorescence with coilin (CB) and DDX6 (PB) antibodies. DAPI staining was used to label nuclei. **B**. Number P-bodies were quantitated using the Fiji platform. **C**. Number and size of Cajal bodies per cell were quantitated using the Fiji platform. **D**. Verification of siRNA efficiency. Western blot verification of knockdown with indicated antibodies.

Figure S7

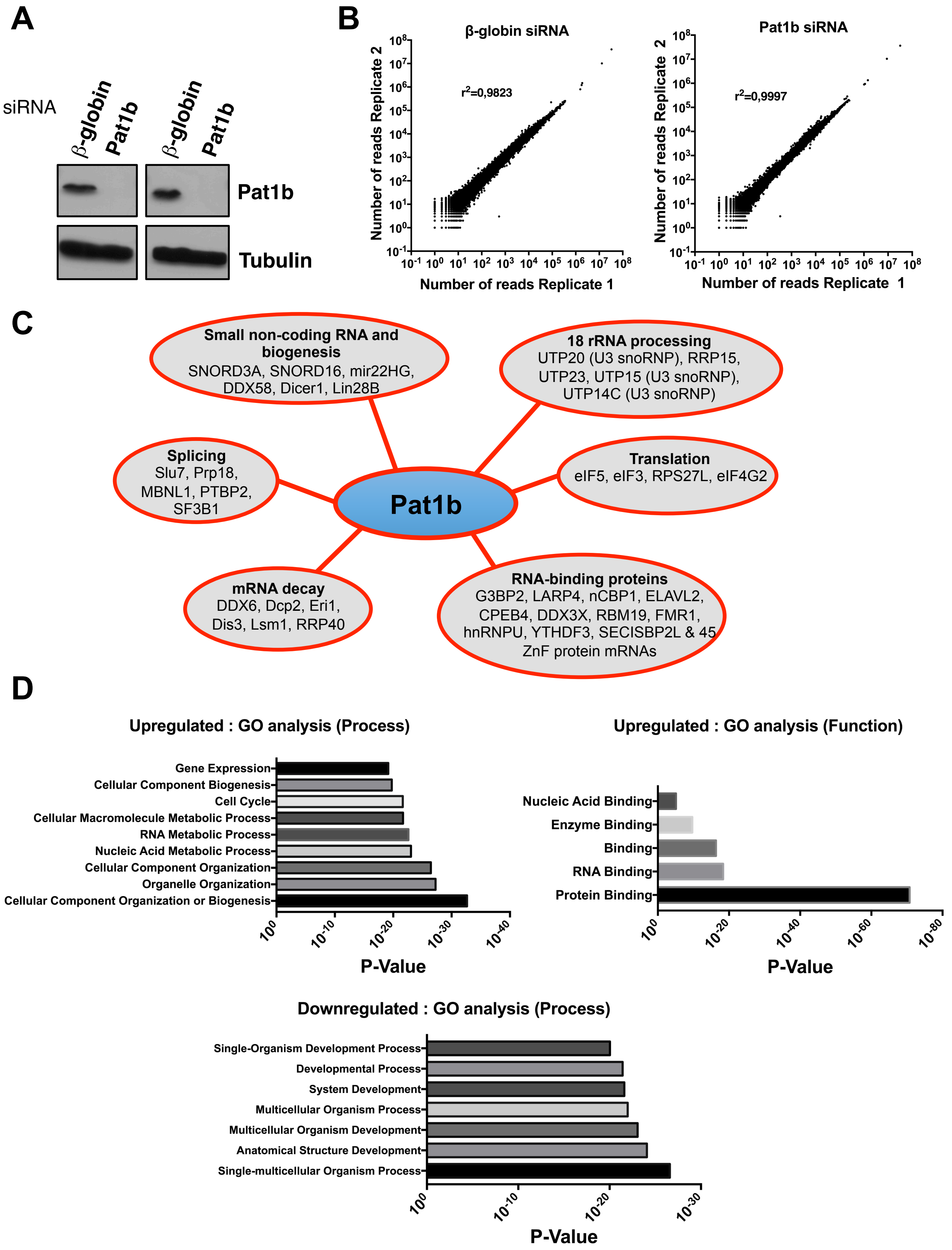

Figure S7. RNAseq analysis. Related to Figure 5.

A. Pat1b was depleted in two independent experiments from HEK293 cells, as verified by western blotting. **B**. RNA extracted from two control and two control Pat1b knock-down sets of cells was sequenced by Illumina NextSeq 500. Paired analysis of the two replicate samples (number of reads per gene) is shown. The correlation coefficient r^2 is indicated. **C**. Schematic cartoon indicating functional classes of up-regulated RNAs. **D**. GO analysis (Process and function) of up-regulated and down-regulated transcripts following Pat1b depletion.

Supplemental Experimental Procedures

Plasmids

The human Pat1b open reading frame (ORF) full length, N-ter, C-ter used in Marnef *et al*., 2012 were sub-cloned into the the N-terminal 3x-FLAG CMV(E7533) 7.1 vector (Sigma Aldrich). The FLAG-GFP construct is described in (Kamenska et al., 2016). The GFP-Pat1b and NES mutant constructs are described in (Marnef et al., 2012). The T522A and T522E mutations were obtained by directed mutagenesis using chimeric PCR and primers with complementary ends. The Flag-SART3 is based on a SART3 construct, a gift of Michael Rape (Song et al., 2010) and was sub-cloned in the N-terminal 3x-FLAG CMV vector as above. The constructs YFP and YFP-Pat1b are described in (Ozgur et al., 2010).

HEK293 cell culture and transfection

HEK293 cells were maintained in DMEM supplemented with 10% fetal bovine serum. Transient transfections were performed as described in (Kamenska et al., 2016). RNAi was carried out using a 2-hit siRNA transfection protocol. On day 1, cells were seeded in 10 cm plates at a density of 2×10^5 cell/ml and immediately transfected with the siRNA. Transfection of siRNA was performed using Lipofectamine 2000, according to manufacturer's instructions, as described for DNA transfection using 15 μg of siRNA per plate. On day 2, the medium was replaced and the second siRNA treatment was performed. On day 3 the medium was replaced and if needed cells were transfected with 10 μg of the appropriate plasmid DNA. Cells were harvested on day 4. The efficiency of RNAi knockdown was assessed by Western blotting.

GFP-Trap based mass spectrometry

HEK293 cells were transiently transfected with YFP and YFP-Pat1b using polyethyleneimine (Polysciences Europe, 1 mg/ml, pH 7.0). 24 hours after transient transfection, cells from one confluent 15-cm dish per transfection were collected and lysed in 1.2 ml ice cold hypotonic lysis buffer (10 mM Tris pH 7.5, 10 mM NaCl, 10 mM EDTA, 0.5% Triton-X100 with freshly added Complete protease inhibitors, Roche). Nuclei were removed by centrifugation at 500 g for 5 minutes at 4°C. The cytoplasmic lysate was incubated with 300 μl GFP-binder magnetic beads ((Rothbauer et al., 2008); Chromotek) for 30 mins at 4°C. Beads were washed 6 times in NET2 buffer (50 mM Tris pH 7.5, 300 mM NaCl, 0.5% Triton-X100). Protein complexes were eluted with 50 μl SDS sample buffer, resolved on 12% polyacrylamide SDS gel and stained with Colloidal Coomassie. Each lane was subdivided into three parts and subjected to HPLC-MS/MS analysis.

Immunoprecipitation and Western blot analysis

Immunoprecipitation of FLAG-tagged proteins was performed with lysates from transfected cells incubated with anti-M2 beads (Sigma Aldrich), and after washing, bound proteins were eluted with SDS loading buffer. When indicated, the transfected lysates were supplemented with RNAse A (Life Technologies) at 40 mg/ml final concentration during the immunoprecipitation binding step. Western blotting analysis was performed with 15% or 20% SDS-PAGE and ECL. Western blots presented in Figures 2 and 3 are representative of 3 independent experiments.

RT-qPCR and RT-PCR

RNA was extracted with TriReagent (Sigma) following manufacturer's instructions and treated with RQ1 DNase I (Promega). RNA (2.5 μg for input and total extract, and the same volume (1/2) of material for immunoprecipitated samples) was converted onto cDNA using oligo(dT), random primers and SuperscriptII (Invitrogen). RT-qPCR was performed using SYBR Green I (Sigma) mix in a Rotor Gene 6000. Results presented in Figures 2D, 3B and 3E are representative of 3 independent experiments.

For conventional RT-PCR, the PCR reactions were performed in 96 well plates using JumpStart™ Taq DNA polymerase (Sigma). PCR products were resolved in a QIAxcel Advanced System (QIAGEN) and percentage of isoform inclusion measured with QIAxcel ScreenGel software.

Endogenous co-immunoprecipitation

Non transfected HEK293 cells were harvested in NET buffer (50mM Tris-HCl, pH 7.5, 150mM NaCl, 0.5% NP40, 1mM EDTA). The lysates were incubated with no antibody, Pat1b or SART3 Antibody for 2h at 4°C. Then the samples were incubated for 3h at 4°C with protein G Sepharose beads. Beads were washed 3 times with TBS (50 mM Tris-HCl, pH 7.5, 150 mM NaCl) and protein complexes were eluted in SDS loading buffer. Western blotting analysis was performed with 15% SDS-PAGE.

Northern blotting and quantitation

After immunoprecipitation reactions, RNAs were recovered from the beads by TriReagent extraction. Purified RNAs were size-fractionated on a 6% denaturing polyacrylamide gel and electroblotted onto a Hybond-N nylon membrane (GE Healthcare). The immobilized RNAs were probed with 5′-terminally labeled sequence-specific oligodeoxynucleotides. The level of snRNAs was quantitated by PhosphorImager.

Subcellular fractionation

HEK293 cells were transfected with FLAG-Pat1b or FLAG-GFP plasmids, and after 24 hours, cells were pelleted in PBS. The cell pellet was resuspended in 500 µl lysis buffer (10 mM HEPES-KOH pH 7.5, 10 mM NaCl, 3 mM $MgCl_2$, 0.35 M sucrose, 0.25% NP40, protease inhibitors) and incubated on ice, 5 min, then centrifuged (2,000 rpm, 5 min, 4 °C). The cytoplasmic supernatant was removed and the pellet washed in lysis buffer lacking NP40, before being resuspended in 250 µl of lysis buffer, with nuclei ruptured with needle and syringe. Western blotting analysis was performed with 15% or 20% SDS-PAGE with equal amounts of cytoplasmic and nuclear proteins, representing 2 % and 8 % of their starting amounts.

HeLa cell immunofluorescence after RNAi

HeLa cells were transfected with siRNA at the time of plating in 6-well plates. After 24 hours, the second hit of siRNA treatment was performed as the cells were plated on 13 mm glass coverslips in 24-well plates. Medium was changed after a further 24 hours and immunofluorescence was performed after 48 hours. Slides were imaged using a Zeiss Axio Observer Z.1 wide-field microscope equipped with a 63x/1.4 Plan-Apochromat objective and a Hamamatsu Orca Flash 4 camera. Briefly, for each slide, a grid of ten regularly spaced positions was defined using ZEN blue and a z-stack (40 slices, 9.36x211x211 µm) was acquired at each position. Processing and analysis were performed with custom-made macros using FIJI (Schindelin et al., 2012). Interphase nuclei were segmented on the basis of the DAPI staining intensity and geometric properties. Cajal bodies were detected by applying a manually-determined threshold to the maximum intensity projection of the coilin channel and identifying positive particles located within nuclei. For P-body segmentation, cell outlines were identified on a combination of the average intensity projections of the DDX6 and DAPI channels. Only domains containing one nucleus were further analyzed. In each, P-bodies were detected by applying a manually-determined threshold to the maximum intensity projection of the DDX6 channel and identifying positive particles with a 0.1-5 μm^2 surface.

Bioinformatic analysis

For gene expression analysis, following trimming using Trim-Galore! to enhance the quality analysed with FastQC (Babraham Bioinformatics), reads were aligned to the full human genome GRCh38 (NCBI) using TopHat (Trapnell et al., 2009). The differential expression between the control and Pat1b-depleted cells was done using DESeq2 (Love et al., 2014) on RStudio with counts generated with HTseq. A paired analysis was performed to obtain fold change with associated adjusted p-value. Gene Ontology analysis using the whole genome was performed using GoTermFinder (Boyle et al., 2004). For splicing analysis, following trimming using Trimmomatic (Bolger et al., 2014) to obtain only reads at 75 bp, reads were alignated to the full human genome as above. Analysis of splicing changes was done using rMATS, using a FDR < 0.1 cut-off for significance and a minimal inclusion level difference of 10% (Shen et al., 2014). For regulated cassette exons properties analysis, data processing was performed with custom scripts written in Perl, with the reference list defined by FDR of > 0.1 and inclusion level [-0.01:0.01]. The two-sided Mann-Whitney test was used to compare properties between datasets (ns : p>0.05, * : p<0.05, ** : p<0,01, *** : p<0.001, **** : p<0.0001). All statistical analysis were performed with RStudio and graphics were

generated with Prism. The splice site strength analysis was performed using the MaxEntScan tool available online (Yeo and Burge, 2014).

RNAseq data was deposited in ArrayExpress, accession number E-MTAB-5577.

siRNAs

β-globin	GGUGAAUGUGGAAGAAGU[dT][dT]
Pat1b	CUAGAAGAUCCAGCUAUUA[dT][dT]
SART3	ACUGCUACGUGGAGUUUAA[dT][dT]
Lsm1	GAAGGACACUUAUAGGCUU[dT][dT]
Lsm2	CCAUUCUGUGGAUCAGUAU[dT][dT]
Lsm8	GGAUGAAAGCCAUGAACGA[dT][dT]

snRNA probes for Northern blot

**U6**	ATCGTTCCAATTTTAGTATA
**U4**	CAAAAATTGCCAATGCCGACTATAT
**U1**	GTCGAGTTTCCCACATTTGGG

snRNA primers for qPCR

U1 Forward	GATACCATGATCACGAAGGTGGTT
U1 Reverse	CACAAATTATGCAGTCGAGTTTCC
U4 Forward	GCGCGATTATTGCTAATTGAAA
U4 Reverse	AAAAATTGCCAATGCCGACTA
U6 Forward	GCTTCGGCAGCACATATACTAAAAT
U6 Reverse	ACGAATTTGCGTGTCATCCTT

Primers for RT-PCR

FASTK Forward	GGACATAGTAGCTGAGGGGTTG
FASTK Reverse	GCTTCTGCCTCAGGTAGCTC
VARS2 Forward	AGCTTCGTCACCCCTTGAT
VARS2 Reverse	CTGCAGATGGGCAGTACCAT
ACOT9 Forward	TCCAGTTTGTGGATGCTCCT
ACOT9 Reverse	TCCAGTTTGTGGATGCTCCT
STRN4 Forward	GGTCGTTGTCGTTGGTGAC
STRN4 Reverse	GGTCAAACTCCAAGGCATTC
CCDC84 Forward	CTCAGATCCGTGAGGTGGAG
CCDC84 Reverse	TGTTCATCCCTTTCCAGCTT

Antibodies

Western blotting
mouse anti-FLAG (Sigma), rabbit anti-SART3 (Bethyl labs), rabbit anti-Prp31 (Bethyl labs), rabbit anti-DDX6 (Bethyl labs), rabbit anti-Lsm1 (Sigma), mouse anti-Lsm2 (Abcam), mouse anti-Lsm8 (SantaCruz), rabbit anti-Pat1b (Bethyl labs), rabbit anti-tubulin (Abcam), rabbit anti-LaminA/C (SantaCruz) and rabbit anti-RPS6 (Cell Signaling).

Immunofluorescence
rabbit anti-DDX6 (Bethyl labs), rabbit (Dundee Cell) or mouse anti-coilin (Abcam), rabbit anti-SART3 (Bethyl labs)

Supplemental References

Bolger, A.M., Lohse, M., and Usadel, B. (2014). Trimmomatic: A flexible trimmer for Illumina Sequence Data. Bioinformatics *btu170*.

Boyle, E.I., Weng, S., Gollub, J., Jin, H., Botstein, D., Cherry, J.M., and Sherlock, G. (2004). GO::TermFinder--open source software for accessing Gene Ontology information and finding significantly enriched Gene Ontology terms associated with a list of genes. Bioinformatics *20*, 3710-3715.

Kamenska, A., Simpson, C., Vindry, C., Broomhead, H., Bénard, M., Ernoult-Lange, M., Lee, B.P., Harries, L.W., Weil, D., and Standart, N. (2016). The DDX6-4E-T interaction mediates translational repression and P-body assembly. Nucleic Acids Res *44*, 6318-6334.

Love, M.I., Huber, W., and Anders, S. (2014). Moderated estimation of fold change and dispersion for RNA-seq data with DESeq2. Genome Biology *15*, 550.

Marnef, A., Weil, D., and Standart, N. (2012). RNA-related nuclear functions of human Pat1b, the P-body mRNA decay factor. Mol Biol Cell *23*, 213-224.

Ozgur, S., Chekulaeva, M., and Stoecklin, G. (2010). Human Pat1b connects deadenylation with mRNA decapping and controls the assembly of Processing-bodies. Mol Cell Biol *30*, 4308-4323.

Rothbauer, U., Zolghadr, K., Muyldermans, S., Schepers, A., Cardoso, M.C., and Leonhardt, H. (2008). A versatile nanotrap for biochemical and functional studies with fluorescent fusion proteins. Mol Cell Proteomics *7*, 282-289.

Schindelin, J., Arganda-Carreras, I., Frise, E., Kaynig, V., Longair, M., Pietzsch, T., Preibisch, S., Rueden, C., Saalfeld, S., Schmid, B*., et al.* (2012). Fiji: an open-source platform for biological-image analysis. . Nature Methods *9*, 676-682.

Sharif, H., and Conti, E. (2013). Architecture of the Lsm1-7-Pat1 complex: a conserved assembly in eukaryotic mRNA turnover. Cell Rep *5*, 283-291.

Shen, S., Park, J.W., Lu, Z.X., Lin, L., Henry, M.D., Wu, Y.N., Zhou, Q., and Xing, Y. (2014). rMATS: robust and flexible detection of differential alternative splicing from replicate RNA-Seq data. PNAS *111*, E5593-5601.

Song, E.J., Werner, S.L., Neubauer, J., Stegmeier, F., Aspden, J., Rio, D., Harper, J.W., Elledge, S.J., Kirschner, M.W., and Rape, M. (2010). The Prp19 complex and the Usp4Sart3 deubiquitinating enzyme control reversible ubiquitination at the spliceosome. Genes Dev *24*, 1434-1447.

Trapnell, C., Pachter, L., and Salzberg, S.L. (2009). TopHat: discovering splice junctions with RNA-Seq. Bioinformatics *25*, 1105-1111.

Yeo, G., and Burge, C.B. (2014). Maximum entropy modeling of short sequence motifs with applications to RNA splicing signals. J Comput Biol *11*, 377-394.

Zhou, L., Hang, J., Zhou, Y., Wan, R., Lu, G., Yin, P., Yan, C., and Shi, Y. (2014). Crystal structures of the Lsm complex bound to the 3' end sequence of U6 small nuclear RNA. Nature *506*, 116-120.
